# Implementation strategies for infection prevention and control promotion for nurses in Sub-Saharan Africa: a systematic review

**DOI:** 10.1186/s13012-019-0958-3

**Published:** 2019-12-30

**Authors:** Amy Elizabeth Barrera-Cancedda, Kathryn A. Riman, Julianna E. Shinnick, Alison M. Buttenheim

**Affiliations:** School of Nursing, University of Pennsylvania, Philadelphia, PA 19104 USA

**Keywords:** Infection prevention and control, Global Health, Nursing, Implementation strategies, Implementation outcomes, Sub-Saharan Africa

## Abstract

**Background:**

Despite impressive reductions in infectious disease burden within Sub-Saharan Africa (SSA), half of the top ten causes of poor health or death in SSA are communicable illnesses. With emerging and re-emerging infections affecting the region, the possibility of healthcare-acquired infections (HAIs) being transmitted to patients and healthcare workers, especially nurses, is a critical concern. Despite infection prevention and control (IPC) evidence-based practices (EBP) to minimize the transmission of HAIs, many healthcare systems in SSA are challenged to implement them. The purpose of this review is to synthesize and critique what is known about implementation strategies to promote IPC for nurses in SSA.

**Methods:**

The databases, PubMed, Ovid/Medline, Embase, Cochrane, and CINHAL, were searched for articles with the following criteria: English language, peer-reviewed, published between 1998 and 2018, implemented in SSA, targeted nurses, and promoted IPC EBPs. Further, 6241 search results were produced and screened for eligibility to identify implementation strategies used to promote IPC for nurses in SSA. A total of 61 articles met the inclusion criteria for the final review. The articles were evaluated using the Joanna Briggs Institute’s (JBI) quality appraisal tools. Results were reported using PRISMA guidelines.

**Results:**

Most studies were conducted in South Africa (*n* = 18, 30%), within the last 18 years (*n* = 41, 67%), and utilized a quasi-experimental design (*n* = 22, 36%). Few studies (*n* = 14, 23%) had sample populations comprising nurses only. The majority of studies focused on administrative precautions (*n* = 36, 59%). The most frequent implementation strategies reported were education (*n* = 59, 97%), quality management (*n* = 39, 64%), planning (*n* = 33, 54%), and restructure (*n* = 32, 53%). Penetration and feasibility were the most common outcomes measured for both EBPs and implementation strategies used to implement the EBPs. The most common MAStARI and MMAT scores were 5 (*n* = 19, 31%) and 50% (*n* = 3, 4.9%) respectively.

**Conclusions:**

As infectious diseases, especially emerging and re-emerging infectious diseases, continue to challenge healthcare systems in SSA, nurses, the keystones to IPC practice, need to have a better understanding of which, in what combination, and in what context implementation strategies should be best utilized to ensure their safety and that of their patients. Based on the results of this review, it is clear that *implementation* of IPC EBPs in SSA requires additional research from an implementation science-specific perspective to promote IPC protocols for nurses in SSA.

## Background

Infectious diseases generate significant morbidity and mortality worldwide [1, 2]. Despite reductions in global prevalence over the last 20 years, a disproportionate amount of the disease burden related to infectious diseases remains in Sub-Saharan Africa (SSA) [3, 4]. Despite impressive reductions in infectious disease burden within SSA [3, 4], communicable illnesses (along with maternal, neonatal, and nutritional causes) comprised 61% of the disability-adjusted life year (DALY) burden in the region as of 2015 [5]. Half of the top ten causes of poor health or death in SSA are infectious diseases [2, 3]. With rapid economic, social, and geographical shifts occurring in the region [6–8], emerging and re-emerging infectious diseases are playing an increasingly important role in the burden of disease. Recent outbreaks like the Ebola viral disease (EVD) epidemics of 2014 and 2018 have highlighted the impact of infectious diseases on healthcare systems and the communities they serve. In the context of already resource-challenged health care systems, the EVD epidemic of 2014 devastated the healthcare infrastructure of three Western African countries [9], and impacted four more [10]. By April 2016, 28,616 confirmed EVD cases and 11,310 deaths were reported for the region [9]; the economic cost of the epidemic was estimated at $3.6 billion, with $2.2 billion in gross domestic product lost in Guinea, Liberia, and Sierra Leone in 2015 [9]. Similar expenditures can be observed for HIV services and care within the region: fiscal requirements for HIV therapy up to 2050 are projected to be as high as 21% and 80% of the GDPs of South Africa and Malawi respectively [11]. Currently, an EVD epidemic in the Democratic Republic of the Congo (DRC) is evolving into a significant public health endeavor, leading to the second largest EVD epidemic in history, with over 500 confirmed cases [12]. Infectious diseases and the damage they can cause to patients, healthcare workers, and health systems remain among the most pressing priorities to be addressed in SSA.

Within the broader category of infectious diseases, healthcare-acquired infections (HAIs) are a major challenge. HAI rates are generally higher in low-income compared to high-income countries [13], with substantial variation across and within countries of all income levels: the cumulative incidence of HAIs ranges from 5.7 to 48.5% within African countries [14]. Traditionally defined, HAIs are infections patients acquire while receiving care in a healthcare facility [15, 16]. Yet, HAIs that impact healthcare workers providing patient care are equally important, especially nurses [17, 18]. While many different types of health care workers (i.e., laboratory technicians, physicians, water and sanitation staff) are at increased risk of acquiring infectious diseases in the healthcare setting, this study focused on nurses for the following reasons: (a) nurses have unique needs (they spend the most amount of time with patients than any other health worker [19] and operate in highly unstandardized and variable circumstances); (b) nurses are by far the largest cadre of healthcare workers in SSA (even though their needs often take second place or are lumped to those of physicians or other healthcare workers).

Two diseases, EVD and tuberculosis (TB), provide excellent exemplars of infectious diseases that disproportionately affect nurses while caring for patients. A total of 718 healthcare worker EVD infections occurred in West Africa, with 396 (55%) confirmed cases among nurses [20]. A combined cumulative incidence rate of EVD among nurses was 43.7 per 1000 in the region, compared to 29.5 and 40.4 per 1000 among physicians and laboratory technicians respectively [20]. Similarly, high rates of TB infections are observed in healthcare workers [21]. The median incidence rate of TB among healthcare workers in SSA was 3871 per 100,000 [21], making the risk of contracting TB among healthcare workers, including nurses, in SSA greater than the risk in the general population in SSA [22]. In terms of HAIs, nurses are often unduly infected, leaving significant burdens on the health system.

Inadequate adherence to infection prevention and control (IPC) standards place millions of patients and healthcare workers, especially nurses, at risk of infectious diseases worldwide, including HAIs. “IPC is a scientific approach and practical solutions to prevent harm caused by infections to patients and health workers” [23]. Effective IPC knowledge and practice are the keystones of a strong healthcare system. The causes of high HAI rates include poor environmental hygiene, inappropriate medical waste disposal, inadequate infrastructure, insufficient equipment, and poor knowledge of infection control protocols all contribute to high HAIs [13].

For example, nurses in SSA may not have enough resources, like biohazard bins or waste bags, to adequately dispose of infectious medical materials [18]. Additionally, nurses may not have access to the following: an adequate healthcare infrastructure to provide safe patient care, familiarity with IPC policies or regulations within their healthcare facilities, and knowledge of effective screening and triage practices to minimize transmission of infectious diseases entering the health facility [18]. The causes of poor IPC places nurses at increased risk of acquiring an infectious disease while serving patients; however, HAIs among healthcare workers and patients are preventable.

The World Health Organization (WHO) [24] has identified a set of evidence-based recommendations on the key components of an IPC program for a national and facility level. These IPC core components include dedicated programs with teams of specialty trained IPC professionals, guidelines, training and education, surveillance, implementation of multi-modal IPC strategies, monitoring/auditing and providing feedback, establishing requirements for workload, staffing, and bed occupancy, and ensuring that the built environment, equipment, and materials are available for IPC practices [24]. These core components are the foundation for the two different branches of IPC precautions: standard precautions and transmission-based precautions [25]. Standard precautions are the basics of the IPC precautions. Used for all patients, this branch of precautions includes hand hygiene practices, appropriate use of personal protective equipment (PPE), respiratory hygiene, appropriate patient placement, injection safety, disinfection, and medical waste disposal [26]. When implemented correctly by health workers, these precautions keep the health worker protected from infection and keep infections from spreading among patients [26]. In addition to standard precautions, the second branch of IPC precautions are transmission-based precautions [26]. The three transmission-based precautions are contact, droplet, and airborne [26]. Contact precautions are used when patients are colonized with an infectious agent and the risk of further transmission is high [26]. For some infectious agents, specialized precautions called administrative precautions are used to further control the spread of infection. Administrative precautions focus on reducing the risk of exposure to patients infected with specific infectious diseases [27]. Administrative control activities include screening, diagnosing, and treating infectious agents [27]. For example, TB is an infectious disease that requires both administrative precautions and transmission-based precautions [27]. Rapidly screening TB suspects via intensive case finding expedites patient diagnosis, which allows for therapy to be expedited as well. Once TB patients are placed on effective therapy, they are no longer infectious to others [28–30].

With sufficient resources to advance health system strengthening initiatives, prevention of infectious diseases, including HAIs, is achievable. Within the global health context, many initiatives have used strategies to incorporate IPC evidence-based practices (EBPs), like standard, transmission-based, and administrative precautions, into standard healthcare practice. Many EBPs are known for many healthcare challenges [31]. EBPs, like hand hygiene, are effective interventions known to reduce infectious agents among patients and healthcare workers [32–34]. Unfortunately, EBPs, including those for IPC, are not effectively implemented in many low- and middle-income countries (LMICs) in SSA [35]. Within implementation science, a variety of implementation strategies have been used to integrate EBPs into clinical practice in LMICs. Proctor et al. [36] defines implementation strategies as “methods or techniques used to enhance the adoption, implementation, and sustainability of a clinical program or practice”. In SSA, many implementation strategies have been used to promote IPC protocols [32, 37–40]. All of these strategies have produced outcomes associated with the original EBP or the strategy utilized. Additionally, implementation outcomes measure the degree in which implementation strategies have been successfully utilized. Implementation outcomes are “the effects of deliberate and purposive action to implement new treatments, practices, and services [41]. In SSA, implementation outcomes have been measured to assess if IPC EBPs have been successfully implemented.

The Conceptual Model of Implementation Research is a framework that outlines the relationships between an EBP, implementation strategies utilized to promote the EBP, and outcomes of the implementation strategies [42]. This framework provides the conceptual underpinnings for the primary research question of this review: how are implementation strategies used to support IPC promotion for nurses in SSA. Using this model, hand hygiene, waste disposal, and correct PPE use are examples of EBPs. Trainings and stakeholder buy-in sessions are examples of implementation strategies, and number of nurses trained and number of trainings conducted are examples of implementation outcomes. Given the burden of infectious diseases in SSA, the promotion of IPC protocols for healthcare workers is critically needed. Yet, limited literature exists on how implementation strategies have been used to advance IPC, for nurses, a commonly overlooked healthcare worker cohort. To address this gap, the purpose of this review is to synthesize and critique what is known about implementation strategies to promote IPC for nurses in SSA.

## Methods

### Search strategy

A systematic approach was used to identify articles from the following databases: PubMed, Ovid/MEDLINE, Embase, Cochrane, and CINHAL. PubMed and Ovid/MEDLINE were selected for their referencing of the biomedical literature. Embase was selected for its focus on international scholarship and a global audience. Cochrane and CINHAL were selected for their reporting on systematic reviews and nursing literature respectively. Two reviewers (AEBC, KAR) independently searched the databases using the search terms for nurses/nursing; IPC, standard precautions or transmission-based precautions; and Sub-Saharan Africa or individual countries in the region. The complete search syntax for each database are included in Additional file 1. As per the recommendations of Whittemore and Knafl [43], the reference sections of each article were reviewed for additional studies meeting eligibility criteria: a methodology known as citation indexing.

**Fig. 1:**
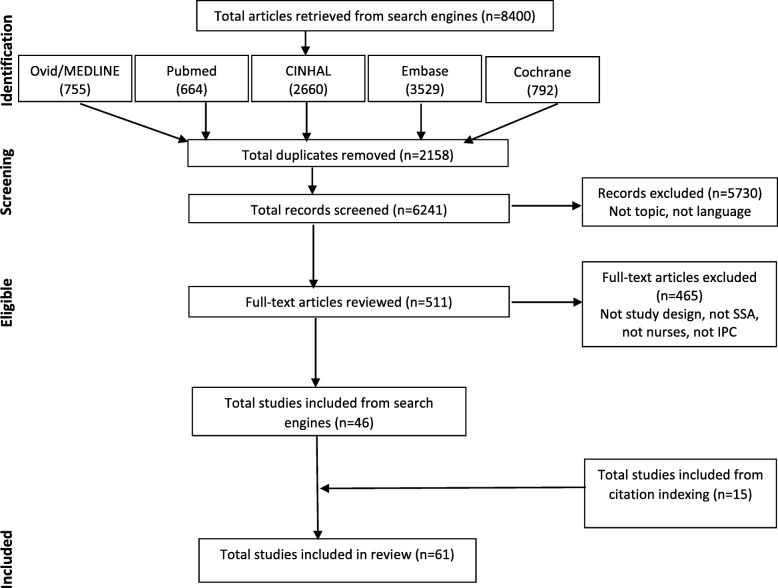
PRISMA flow diagram for search strategy

Figure 1 depicts the Preferred Reporting Items for Systematic Reviews and Meta-Analyses (PRISMA) flow diagram used to report study findings [44]. The protocol for this review was not registered.

### Eligibility criteria

Inclusion and exclusion criteria were developed to identify research that empirically evaluated or tested implementation strategies to promote IPC protocols for nurses in SSA. Studies were eligible for inclusion if written in English, peer-reviewed, published between 1998 and 2018, implemented in SSA, targeted nurses, and promoted IPC EBPs. All study designs (RCT, cross-sectional, cohort, qualitative) were eligible. Studies were excluded if they did not meet the aforementioned criteria. For example, non-empirical studies (reviews, commentaries, briefs, editorials, reports, guidelines) and studies that did not specifically evaluate or test an implementation strategy (e.g., prevalence studies, modeling studies) were excluded from this review.

### Study selection and data extraction

Titles and abstracts identified in the initial search were de-duplicated and then screened using inclusion and exclusion criteria for full-text review. Citation-index searching was conducted on articles that met inclusion criteria following full-text review, and eligible articles were similarly screened. The following implementation-related data elements were extracted from studies included in the review: author, year of publication, purpose, country, study design, IPC EBPs, implementation strategies, and implementation outcomes.

For each article, implementation strategies and outcomes were categorized based on the definitions produced by Powell et al. [45] and Proctor et al. [41] respectively. Further categorization of implementation outcomes based on their association with the EBPs or implementation strategies to promote the EBPs was conducted. For example, hand hygiene is an EBP. An educational training (implementation strategy) may be provided to nurses to promote the uptake of hand hygiene practices. The outcomes of this initiative may be related to the EBP and/or to the training. An example of a possible outcome related to the above scenario for the EBP is penetration (i.e., increased number of nurses practicing hand hygiene out of total number of nurses). An example of a possible outcome associated with the implementation strategy (training) is acceptability (i.e., 95% of nurses agreed the training material on hand hygiene was good, informative, and useful). Two reviewers (AEBC, KAR) screened all articles for inclusion/exclusion. After all articles were screened and deemed to meet the inclusion criteria of the review, data extraction was then conducted by the primary reviewer (AEBC).

### Quality screening

Each included article was assessed for quality using tools developed by the Johanna Briggs Institute (JBI). JBI focuses on the promotion of evidence-based practices through effective healthcare measures to a global community [46]. Implementation and translational science are key methodologies embraced and promoted by JBI [46], making these appraisal criteria particularly well-suited to the purpose and scope of this review.

JBI uses individual assessment tools for each type of study (randomized control trial, cohort, case-study, qualitative investigation, etc.) to assess quality. Study designs that had global cut-off criteria were assessed using that criteria. For studies where no global criteria existed, the reviewers established pre-determined cut-off scores before the appraisal process was initiated. Each JBI tool has specific questions addressing bias, confounding variables, statistical analyses, methodological validity, and outcome reliability [47]. Table 1 contains the quality appraisal and scoring criteria utilized in this review.

**Table 1:** Quality appraisal summary and criteria

Study design	Tool	Response options	Scoring	Cut-off score
RCT [47]	MAStARI	Yes, No, Unclear, Not applicable	Yes = 1; No/Unclear/NA = 0	6
Quasi-experimental [47]	MAStARI	Yes, No, Unclear, Not applicable	Yes = 1; No/Unclear/NA = 0	5
Cross-sectional [47]	MAStARI	Yes, No, Unclear, Not applicable	Yes = 1; No/Unclear/NA = 0	5
Qualitative [48]	QARI	Yes, No, Unclear, Not applicable	Yes = 1; No/Unclear/NA = 0	5
Mixed methods [49]	MMAT	Yes, No, Cannot Tell	Yes = 1; No/Cannot Tell = 0	50%

For quantitative studies, the JBI-Meta Analysis for Statistics Assessment and Review Instrument (MAStARI) was used. For qualitative studies, the JBI-Qualitative Assessment and Review Instrument (QARI) was used. For mixed methods studies, JBI does not have an appraisal tool. The mixed methods appraisal tool (MMAT) produced by Pluye et al. [49] at McGill University was used. This tool has specific quantitative, qualitative, and integration (mixed methods) questions to assess study quality. Two reviewers (AEBC, KAR) appraised all studies for quality, and an additional reviewer (JES) appraised a random sample of 20% (*n* = 20) of the articles. Any discrepancy in the appraisal process was discussed by all reviewers and consensus was reached.

## Results

### Database and citation indexing search results

The initial database search produced 8400 results. Further, 2159 duplicates were removed. Of the 6241 studies that remained, 511 met the criteria for full-text review. Forty-six studies met the inclusion criteria. Citation indexing yielded an additional 15 studies that met the inclusion criteria. A total of 61 studies have been included in this review (see Fig. 1 for PRISMA flow diagram) [32–34, 38–40, 50–104].

Table 2 provides a summary of the individual study characteristics of the studies included in this review. Table 3 provides a table of evidence of the studies in the review. The majority of the studies were conducted in South Africa (*n* = 18, 30%), within the last 18 years (*n* = 41, 67%), and utilized a quasi-experimental design (*n* = 22, 36%). After South Africa, the other countries where the majority of studies were conducted were Nigeria (*n* = 5, 8%), Kenya (*n* = 4, 7%), and Zambia (*n* = 4, 7%). The majority of studies in this review focused on HIV (*n* = 24, 39%), TB (*n* = 6, 10%), Ebola (*n* = 6, 10%), or did not focus on a specific disease or infection (*n* = 14, 23%). The non-disease studies focused on standard precautions.

**Table 2:** Study characteristics (*N* = 61)

Characteristic	Number of studies (*n*)	Percentage of studies (%)
Sub-Saharan country		
South Africa	18	29.5
Nigeria	5	8.2
Kenya	4	6.6
Zambia	4	6.6
Rwanda	4	6.6
Uganda	4	6.6
Ethiopia	3	4.9
Cameroon	3	4.9
Tanzania	2	3.3
Malawi	2	3.3
Sierra Leone	2	3.3
Liberia	2	3.3
Mali	1	1.6
Sudan	1	1.6
Eritrea	1	1.6
Democratic Republic of the Congo	1	1.6
Guinea	1	1.6
Swaziland	1	1.6
Zimbabwe	1	1.6
Botswana	1	1.6
Publication date		
1998–2003	3	4.9
2004–2009	17	27.9
2010–2015	21	34.4
2015+	20	32.8
Study design		
RCT	7	11.5
Quasi-experimental	22	36.1
Cohort	13	21.3
Cross-sectional	12	19.7
Mixed methods	3	4.9
Qualitative	3	4.9
Multi-modal	1	1.6
Healthcare Workers		
Healthcare workers (physicians, nurses, laboratory techs, etc.)	47	77.0
Nurses only	14	22.9
Diseases/infections		
HIV	24	39.3
TB	6	9.8
Ebola	6	9.8
Multiple diseases	3	4.9
Malaria	2	3.3
CLABSI	1	1.6
VAP	1	1.6
SSI	1	1.6
Bloodborne (non- CLABSI)	1	1.6
STIs (non-HIV)	1	1.6
None (hand hygiene, disinfection focused)	14	23.0
Evidence-based practices		
Standard Precautions	21	34.4
Multiple SP	11	18.0
Hand hygiene	8	13.1
Disinfection	1	1.6
Waste management	1	1.6
Transmission-based precautions	4	6.6
PPE	1	1.6
Isolation/quarantine	1	1.6
Immunization	1	1.6
Post-exposure prophylaxis	1	1.6
Administrative precautions	36	59.0
Treatment	21	34.4
Screening/surveillance	11	18.0
Diagnosis	4	6.6
Quality assessment score		
MAStARI 4	1	1.6
MAStARI 5	19	31.1
MAStARI 6	10	16.4
MAStARI 7	16	26.2
MAStARI 8	5	8.2
MAStARI 9	3	4.9
MAStARI 10	1	1.6
MAStARI 11	2	3.3
QARI 5	1	1.6
QARI 6	0	0.0
QARI 7	1	1.6
QARI 8	2	3.3
MMAT 0%	0	0.0
MMAT 50%	2	3.3
MMAT 75%	1	1.6
MMAT 100%	0	0.0

**Table 3:** Table of evidence

No.	Author/Year	Purpose	Country	Study design	Participants	Disease	EVB IPC intervention	Quality
1	Allegranzi et al. [32]	To assess the feasibility and effectiveness of WHO hand hygiene improvement strategy in low-income country	Mali	Pre/Post Design	224 Healthcare workers (# of nurses not specified)	NA	Hand hygiene/washing	MAStARI 5
2	Brown et al. [50]	To evaluate the impact, acceptability, and feasibility of a novel toolkit to prevent HIV	Kenya	Mixed methods	10 HCWs (2 nurses) 40 HIV Patients (20 HIV+ women; 10 discordant couples)	HIV	HIV prevention/safe conception practices associated with HIV prevention	MMAT 50%
3	Brown et al. [51]	To assess the impact of an immunization training on knowledge and practice of HCWs	Nigeria	Pre-Post experimental design/RCT study	69 HCWs (17 were nurses/midwives/CNO)	NA	Immunization	MAStARI 9
4	Courtenay-Quirk et al. [52]	To identify key factors in bloodborne Pathogen Exposure (BPE) incidence, report, and Post-exposure prophylaxis (PEP) uptake to inform the development of a multi-component intervention strategy	Botswana Zambia Tanzania	Mixed methods	HCWs (2851 for all 3 countries; # of nurses not specified)	Any blood- borne pathogen	Post-exposure prophylaxis (PEP)	MMAT 50%
5	Dahinten et al. [53]	To describe the implementation and feasibility of the “Pratt Pouch,” a pre-packaged ARV medicated foil package for infants born to HIV+ mothers in non-healthcare facilities	Zambia	Pre/Post Design	41 HCWs (16 nurse, 18 community health-care workers, and 7 pharmacists) 150 HIV+ pregnant women	HIV	ART (HIV therapy)	MAStARI 7
6	Durrheim et al. [54]	To study a novel surveillance system to address deficiencies in identifying infectious disease syndromes	South Africa	Prospective cohort study	32 Nurses	Multiple (Polio, Cholera, Measles, Plague, Meningococcal disease, Yellow fever, Dysentery, Viral hemorrhagic fevers)	Surveillance/screening	MAStARI 8
7	Elnour et al. [39]	To assess nursing and sanitation staff knowledge and practice regarding health care waste management	Sudan	Pre/Post Design	200 HCWs (# of nurses not specified) 100 HCWs received training intervention; 100 HCWs were controls	NA	Waste management and proper disposal	MAStARI 9
8	Farley et al. [55]	To develop and evaluate a nurse case management model and intervention using the tenants of the Chronic Care Model (CCM) to manage MDR-TB treatment for patients	South Africa	Pre/Post Design	1 nurse case manager 40 MDR-TB patients	MDR-TB HIV	MDR-TB treatment/monitoring	MAStARI 5
9	Fatti, G [56].	To evaluate the effectiveness of a Quality Nurse Mentor (QNM) health systems strengthening intervention to improve PMTCT processes and outcomes	South Africa	Pre/Post Design	Number of nurses not directly stated All pregnant women attending material health facilities were eligible to enroll in intervention (specific #s of samples not specified)	HIV	PMTCT (HIV therapy)	MAStARI 5
10	Gous et al. [57]	To assess the feasibility and accuracy of the implementation of a nurse-operated multiple POCT in 2 ART clinics	South Africa	Cross-sectional study	3 Senior level nurses 793 HIV+ patients from 2 clinical sites	HIV	Point-of-care testing (POCT)	MAStARI 7
11	Holmen et al. [58]	To improve hand hygiene (HH) compliance among physicians and nurses using the WHO’s guidelines	Rwanda	Pre/Post Design	HCWs (54 nurses; 12 physicians)	N/A	Hand hygiene (hand washing)	MAStARI 5
12	Holmen et al. [33]	To assess the impact of hand hygiene (HH) programs aimed at improving compliance AND to identify unique challenges to HH sustainability	Rwanda	Pre/Post Design	HCWs(56 nurses; 11 physicians)	N/A	Hand hygiene (hand washing)	MAStaARI 5
13	Howard et al. [59]	To evaluate the effectiveness and acceptability of a combination intervention package designed to improve isoniazid preventative therapy (IPT) initiation, adherence, and completion among PLHIV	Ethiopia	Mixed methods randomized cluster trial (RCT) (randomization occurred at the clinic level, not individual)	HCWs (10 nurses; 2 health officers; 9 peer educators) # of patients not specified; 10 out of 11 sites were selected for participation (patients pulled from these sites)	TB	IPT (TB therapy)	MAStARI 9
14	Imani et al. [60]	To evaluate the effectiveness and cost-effectiveness of 2 interventions: on-site support (OSS) and integrative management of infectious disease (IMID) for HCWs	Uganda	Mixed methods randomized cluster trial (RCT) (randomization occurred at the clinic level, not individual)	Mid-level professionals (including 20 nurses, 48 clinical officers, registered midwives) 2 MLP per site = 72 MLPs 687 total patients	Malaria, TB, HIV, and other childhood infectious diseases	Screening, diagnosis, therapy	MAStARI 8
15	Jere et al. [61]	To assess the effects of a peer-to-peer intervention on rural HCWs universal precautions and client teaching	Malawi	Pre/Post Design	HCWs (clinicians, including nurses, and technicians; clinical support workers; non-clinical workers (no specific # of nurses)	HIV	Universal precautions AND HIV prevention associated with universal precautions	MAStARI 8
16	Jones et al. [62]	To describe the educational and transcultural strategies employed to bridge the gap between IPC policy and clinical practice of HCWs	Tanzania	Qualitative study	HCWs (# nurses not specified)	NA	Standard Precautions/UP	QARI 8
17	Jones-Konneh et al. [63]	To reveal the importance and effect of intensive education of HCWs during an Ebola (EVD) outbreak	Sierra Leone	Cross-sectional study	HCWs (# nurses not specified)	Ebola	Standard precautions and transmission- based precautions for EVD prevention	MAStARI 5
18	Kaponda et al. [64]	To evaluate the impact of a peer group intervention on work-related knowledge and behavior on universal precautions among HCWs	Malawi	Pre/Post Design	Roughly 561 HCWs (clinicians, including nurses, and technicians; clinical support workers; non-clinical workers (no specific # of nurses) 678 Patients	HIV	Universal precautions AND HIV prevention associated with universal precautions	MAStARI 5
19	Karari et al. [65]	To evaluate the uptake, acceptability, and effectiveness of Uliza: a telephone consultation service for HCWs	Kenya	Prospective cohort study	296 HCWs (188 physicians, 66 nurses, 23 medical officers. 2 pharmacy technicians, and 17 other)	HIV	ART (HIV therapy)	MAStART 7
20	Kerrigan et al. [66]	To explore the feasibility and acceptability of three active case finding strategies for TB to inform their optimal implementation in a larger, randomized, cluster trail	South Africa	Qualitative study	25 participants (10 HCWs (# nurses not specified), 8 TB patients, and 7 family members of TB patients)	TB	TB screening/active case finding	QARI 8
21	Kunzmann et al. [67]	To develop and implement an evidence-based bundle of care (Best Care Always) to prevent pediatric VAP	South Africa	Prospective cohort study	HCWs (doctors and nurses) (# of nurses not specified)	VAP	“Best Care Always:” an evidence- based HCAI prevention bundle	MAStARI 6
22	Labhardt et al. [68]	To assess the availability of equipment and staff knowledge of PMTCT	Cameroon	Pre/Post Design	HCWs (no denominator provided; study states 42% were staff nurses; 40% were registered nurses; 18% care assistance among nurse-lead facilities (physicians were also included in this study)	HIV	PMTCT (HIV therapy)	MAStARI 5
23	Levy et al. [69]	To describe a successful partnership to implement a national IPC training and PPE supply program in all Primary Health Units (PHUs)	Sierra Leone	Cross-sectional study	4264 HCWs (# of cadres not specified)	Ebola	Standard precautions and transmission- based precautions for EVD prevention	MAStARI 6
24	Lewin et al. [70]	To assess whether adding a training intervention for clinic staff to usual DOTS strategy for TB would affect TB treatment outcomes	South Africa	Clustered RCT (randomization occurred at the clinic level; total number of clincis-24 (with 50 patients per clinic)	HCWs (doctors, nurses, educators, clerical staff, CHWs) # of nurses not specified Roughly 1200 patients	TB	TB therapy	MAStARI 11
25	Liautaud et al. [71]	To examine the effectiveness of a 1-year certificate program in IPC and occupational health (OH) aimed at empowering HCWs to act as change agents for improving workplace-based HIV and TB prevention	South Africa	Mixed methods	32 HCWs (56% were nurses)	HIV TB	HIV/TB IPC	MMAT 75%
26	Liu et al. [72]	To describe the Chinese response to the Ebola epidemic in Liberia	Liberia	Cross-sectional study	HCWs (nurses, social workers, cleaners, and technicians) # nurses not specified	Ebola	Ebola ETU safe design/layout for patient isolation and infection reduction; standard precautions; transmission-based precautions	MAStARI 7
27	Mahomed et al. [73]	To evaluate infection control in intensive care units (ICUs) using the Infection Control Assessment Tool (ICAT)	South Africa	Cross-sectional study	Nurses (# not specified)	NA	IPC Practices (including standard precautions and transmission-based precautions)	MAStARI 6
28	Miceli et al. [74]	To describe the Integrated Infectious Disease Capacity Building Evaluation approach to integrating advances in health professional educational theory in the context of primary healthcare system	Uganda	Pre/Post Design	72 HCWs (clinical officers, nursing officers) # of nurses not specified (36 sites total)	Infectious Diseases (emphasis on HIV, TB, and malaria)	HIV prevention/ART, TB screening/therapy, malaria screening/therapy	MAStARI 7
29	Mbombo & Bimerew [75]	To evaluate students’ clinical performance on PMTCT competencies integrated into the standard nursing curriculum and to determine the effectiveness and relevance PMTCT training program	South Africa	Pre/Post Design	154 student nurses	HIV	PMTCT (HIV therapy)	MAStARI 5
30	Ogoina et al. [76]	To describe the experiences of a tertiary teaching hospital in preparing and responding to the 2014 EVD outbreak	Nigeria	Multi-modal	HCWs (70 doctors, 61 nurses, and 59 other medical staff)	Ebola	Standard precautions and transmission-based precautions for EVD prevention	MAStARI 7 QARI 7
31	Otu et al. [77]	To assess the effect of using a tablet computer application to deliver an educational intervention to change HCWs EVD-related knowledge and attitudes	Nigeria	Pre/Post Design	203 HCWs (94 CHWs, 26 nurses, 8 lab staff, and 75 other	Ebola	Standard precautions and transmission-based precautions for EVD prevention	MAStARI 5
32	Parker et al. [78]	To describe the results of an evaluation of the impact of a low-cost, brief nursing intervention on the utilization of Safe Water System (SWS) and knowledge of proper hand-washing	Kenya	Pre/Post Design	11 Nurses	NA	Safe Water System (Hand hygiene)	MAStARI 5
33	Richards et al. [79]	To describe the implementation and impact of a bundle to reduce CLASI in the Netcare group of private hospitals	South Africa	Prospective cohort study	HCWs (# nurses not specified)	NA	“Best Care Always:” an evidence- based HCAI prevention bundle	MAStARI 7
34	Samuel et al. [80]	To examine the feasibility of adherence to quality standards once established, with reference to hand washing practice as a measure of infection prevention	Eritrea	Qualitative study	HCWs (10 physicians, 10 nurses, 14 health assistants and support staff) 30 patients	NA	Hand hygiene (hand washing)	QARI 8
35	Schmitz et al. [81]	To define baseline rates of HCW hand hygiene adherence and assess the impact of implementing the WHO Multi-modal Hand Hygiene Strategy at an academic hospital	Ethiopia	Pre/Post Design	1000 HCWs (505 physicians, 291 medical students, 144 nurses, and 60 other)	NA	Hand hygiene/washing	MAStARI 5
36	Shumbusho et al. [82]	To evaluate the results of a pilot program of nurse-centered ART	Rwanda	Retrospective cohort study	3 Nurses (at 3 health centers)	HIV	ART (HIV therapy)	MAStART 7
37	Speare et al. [83]	To describe the results of a training strategy for communicable disease control nurses	South Africa	Cross-sectional study	20 Nurses	NA	Surveillance/screening	MAStARI 6
38	Tillerkeratne et al. [84]	To determine whether a multi-faceted intervention targeting health care personnel would reduce CAUTI rate in a public hospital	Kenya	Pre/Post Design	Roughly 44 HCWs (nurses and clinical officers) # nurses not specified	NA	CAUTI Infection Prevention and Control	MARtARI 5
39	Uneke et al. [34]	To promote the adoption of the WHO HH guidelines to enhance compliance among doctors and nurses and improve patient safety in a teaching hospital	Nigeria	Cross-sectional study	202 HCWs (39 physicians and 163 nurses)	NA	Hand hygiene/washing	MAStARI 6
40	Uneke et al. [85]	To assess the impact of a stethoscope disinfection campaign among doctors and nurses	Nigeria	Pre/Post Design	HCWs (39 physicians and 163 nurses)	NA	Disinfection (of medical equipment)	MAStARI 5
41	Van Rie et al. [86]	To evaluate the implementation of three models of provider-initiated HIV counseling and testing for TB patients	Democratic Republic of the Congo	Cross-sectional study	10 HCWs (6 research nurses, 2 VCT staff, 1 counselor/nurse, 1 TB nurse (1238 patients at 3 TB clinics)	HIV TB	HIV Counseling/Testing AND TB Prophylaxis	MAStARI 7
42	Wanyu et al. [87]	To describe the introduction, successes, and challenges implementing a PMTCT program using trained birth attendants	Cameroon	Cross-sectional study	30 Trained Birth Attendants (42 mother-newborn dyads)	HIV	PMTCT (HIV therapy)	MAStARI 7
43	Welty et al. [88]	To describe how the Cameroon Baptist Convention Health Board (CBHHB) successfully integrated PMTCT into routine antenatal care	Cameroon	Cross-sectional study	690 Nurse, midwives, nurse aids, and trained birth attendants	HIV	PMTCT (HIV therapy)	MAStARI 7
44	White et al. [89]	To evaluate three different methods of checklist training by assessing change in behavior at 3–6 months post training on the WHO Surgical safety checklist	Guinea	Cross-sectional study	HCWs (4 surgeons, 7 anesthetists, and 2 ward nurses)	NA	IPC Practices during surgeries (disinfection of equipment, decontamination of equipment, disinfection of environment)	MAStARI 6
45	Xi et al. [90]	To investigate the importance of supervision through video surveillance in improving the quality of personal protection in preparing health care workers working in Ebola treatment units	Liberia	Cross sectional study	HCWs (23 physicians; 8 nurses)	Ebola	PPE use	MAStARI 7
46	Zaeh et al. [91]	To evaluate the impact of a cost-effective quality improvement intervention targeting active TB cases and provision of IPT among those without active TB disease	Ethiopia	Pre/Post Design	4 HCWs (2 physicians; 2 nurses) 751 HIV+ patients	HIV TB	TB screening/IPT Prophylaxis	MAStARI 5
47	*Bedelu et al. [92]	To describe how the integration of HIV care and treatment into primary health care in Lusikisiki overcome some of the challenges of working in a resource-limited rural area, to achieve good treatment outcomes and clinical outcomes	South Africa	Prospective cohort study	HCWs (nurses and community health workers) (# of nurses not specified) 2200 patients	HIV	ART (HIV therapy)	MAStARI 7
48	Elden et al. [38]	To implement and evaluate a program of intensive case finding for TB into a high HIV prevalence, low resource, rural setting	Swaziland	Prospective cohort study	HCWs (nurses and HIV counselors) (# of nurses not specified) 1467 HIV patients	TB	TB screening/intensive case finding	MAStARI 6
49	Charalambous et al. [93]	To evaluate the feasibility and acceptability of a specialist clinical service for HIV-infected mineworkers	South Africa	Prospective cohort study	HCWs (physicians, nurses) (3 nurses) 1773 patients	HIV TB	ART (HIV therapy) TB screening/INH therapy	MAStARI 6
50	Fairall et al. [40]	To assess the effects on mortality, viral suppression, and other health outcomes and quality indicators of the Streamlining Tasks and Roles to Expand Treatment and Care for HIV (STRETCH) program	South Africa	Clustered RCT (randomization occurred at the clinic level; total number of clincis-31	103 nurses (this number represents the number of nurses trained in the intervention group)	HIV	ART (HIV therapy)	MAStARI 10
51	Harrison et al. [94]	To evaluate the implementation of syndrome packets and healthcare worker training of sexually-transmitted diseases	South Africa	Clustered RCT (randomized at the clinic level; total of 10 clinics)	5 nurses (one from each intervention clinic)	Sexually- transmitted diseases	STD Syndromic case management	MAStARI 11
52	Naidoo et al. [95]	To measure knowledge changes among HCWs who participated in a TB training program and make recommendations for future TB trainings	South Africa	Pre/Post Design	267 HCWs (physicians, nurses, and other HCWs) (171 nurses, # of physicians or other HCWs not specified)	TB	TB diagnosis, treatment, and treatment monitoring	MAStARI 5
53	Morris et al. [96]	To describe experiences with task-shifting in Lusaka in a large public sector ART program	Zambia	Prospective cohort study	HCWs (Clinical officers, which practice as NPs, nurses, and peer educators) 71,000 patients	HIV	ART (HIV therapy)	MAStARI 6
54	Perez et al. [97]	To report on activities and lessons learned during the first 18-months of a rural program of PMTCT of HIV	Zimbabwe	Prospective cohort study	20 nurses and midwives 2308 patients	HIV	PMTCT (HIV therapy)	MAStARI 7
55	Sanne et al. [98]	To assess the efficacy of “doctor-initiated-nurse-monitored” ART to doctor-initiated-doctor-monitored” ART using a composite endpoint reflecting both treatment outcomes and patient management	South Africa	RCT	HCWs (2 physicians, 2 nurses) (812 patients)	HIV	ART (HIV therapy)	MAStART 7
56	Ssekabira et al. [99]	To evaluate the impact of a training on the quality case management in 8 health facilities roughly one-year after the implementation of artemether-lumefantrine as a recommended 1st-line treatment regimen	Uganda	Pre/Post Design	170 HCWs at 8 sites (each site had 1 MO, 2 CO, 5 nurses, 5 midwives, 4 nursing assistants, 1 dental officer, 1 lab tech, 1 lab assistant, 1 records officer, 1 educator, 1 health assistant) (Roughly 112 nurses) 76,705 patients	Malaria	Malaria screening, diagnosis, treatment	MAStART 5
57	Sserwanga et al. [100]	To describe the impact of a sentinel site malaria surveillance system promoting laboratory testing and rational antimalarial drug use	Uganda	Prospective cohort study	HCWs AT 6 sites (each site had 1 MO, 2 CO, 5 nurses, 5 midwives, 4 nursing assistants, 1 dental officer, 1 lab tech, 1 lab assistant, 1 records officer, 1 educator, 1 health assistant) (Roughly 84 nurses) 424,701 patients	Malaria	Malaria screening/surveillance/ intensive case finding	MAStARI 7
58	Stringer et al. [101]	To report the feasibility and early outcomes of scaling up an ART program	Zambia	Prospective cohort study	HCWs (# nurses not specified) 21,755 patients	HIV	ART (HIV therapy)	MAStARI 8
59	Umulisa et al. [102]	To describe a multi-strategy intervention with a focus on ensuring stable water supply to improve hand hygiene compliance in a district hospital	Rwanda	Pre/Post Design	HCWs (physicians, nurses, student nurses)	NA	Hand hygiene/washing	MAStARI 5
60	Driessche et al. [103]	To develop and evaluate training materials for provider-initiated HIV counseling, testing, prevention involved in care of patients with TB at the primary health care clinic level	Democratic Republic of the Congo	Pre/Post Design	65 HCWs completed post-test assessment (7 physicians, 38 nurses; 16 lab techs, 4 district supervisors)	HIV	HIV Counseling/Testing AND TB testing/therapy	MAStARI 5
61	Workneh et al. [104]	To report on the effectiveness of a clinical mentoring program at decentralized ART sites dedicated to promoting the scale-up of quality pediatric HIV care and treatment	Botswana	Retrospective cohort study	HCWs (physicians, nurses) (# of nurses not specified) 374 patient charts	HIV	ART (HIV therapy)	MAStARI 6

### Healthcare worker cadres

This review was conducted to investigate what is known about implementation strategies utilized to promote IPC protocols for nurses. Forty-seven (77%) studies included healthcare worker samples, including physicians, pharmacists, laboratory technicians, nurse aids, trained birth attendants, residents, and nurses. Fewer studies (*n* = 14, 23%) had sample populations comprising nurses only.

### IPC evidence-based practices

A variety of EBPs were represented in this review. The majority of studies focused on administrative precautions (*n* = 36, 59%). Standard precautions and transmission-based precautions represented forty-one (34%) and four (7%) studies respectively. Among the administrative precautions, treatment was the most frequently reported EBP (*n* = 21, 34%), followed by screening (*n* = 11, 18%) and diagnosis (*n* = 4, 7%). Studies focusing on HIV treatment or TB screening comprised the majority of studies in this section.

Only four (6%) studies focused on transmission-based precautions. Each study reported on a unique precaution. Transmission-based studies focused on correct PPE use, appropriate patient placement, immunization, and post-exposure prophylaxis.

Studies that addressed standard precautions did so by incorporating multiple precautions (*n* = 11, 18%) or focused on hand hygiene only (*n* = 8, 13%). Other studies that focused on standard precautions addressed medical equipment disinfection or appropriate waste management.

### Implementation strategies

The most frequent implementation strategies used to promote IPC protocols in included studies were education (*n* = 59, 97%), quality management (*n* = 39, 64%), planning (*n* = 33, 54%), and restructure (*n* = 32, 53%). A variety of educational strategies were used to promote IPC protocols for nurses. Strategies used included didactic lectures, simulations, on-site mentorship, visual reminders, and demonstrations. Quality management strategies generally consisted of audit and feedback sessions provided to nurses in real time to promote the uptake of an IPC EBP. Planning strategies consisted of collaborations, partnerships, or buy-in sessions that were established at a higher administrative level for nurse IPC involvement. Planning strategies were mostly utilized in conjunction with other strategies. For example, a partnership between the government of a country and an academic institution may be formalized to provide nurses training or mentorship on IPC protocols. Planning strategies were also used to inquire about nurses’ experiences with IPC EBPs. A variety of restructure strategies were used to promote IPC. Nurses may be task-shifted to include IPC activities within their scope of work, provided additional resources (i.e., alcohol-based hand gel) to make adhering to IPC protocols easier, or promoted to a higher nursing position whereby IPC became the focus of the new role. Only eight (*n* = 8, 13%) studies used financing incentives to promote IPC protocols among nurses. When used, a financial strategy was generally associated with external funders providing resources to initiate an EBP intervention or providing over-time compensation to healthcare workers. For example, the Elizabeth Glaser Pediatric AIDS Foundation, the Axios Foundation, and the Boehringer Ingleheim Pharmaceutical Company provided funds to initiate an antiretroviral therapy (ART) initiation program for HIV patients in Cameroon [88]. Zero (0%) policy strategies were used.

An exhaustive list of implementation strategies is provided for each study in Table 4. Bolded strategies targeted nurses. In order to be included in this review, studies needed to report the use of implementation strategies for nurses. Many studies used implementation strategies that targeted patients, non-nurse healthcare workers, or aspects of the healthcare system. Few studies used implementation strategies solely for nurses. A summary of implementation strategies used for nurses and non-nurses (i.e., patients or a healthcare system-specific matter) is provided in Table 5. Most studies used education (*n* = 58, 95%) for nurses; a drastic contrast to the number of studies that used education (*n* = 1, 2%) for non-nurses. Other discordant results between strategies used for nurses compared to strategies used for non-nurses included planning, restructure, and finance. These strategies were used more for non-nurses than nurses. Twenty-one (34%), 25 (41%), and six (10%) studies used planning, restructure, and finance strategies respectively for non-nurses. Planning, restructure, and finance were used in 12 (20%), seven (12%), and two (3%) studies respectively for nurses.

**Table 4:** List of Implementation Strategies and Outcomes Produced in each Study (*n* = 61)

No.	Author/ Year	EVB IPC intervention	Implementation strategy*	Implementation outcome for the EBP*	Implementation outcome for the Implementation strategy*
1	Allegranzi et al. [32]	Hand hygiene/washing	1. PLAN: (conducted local consensus discussions with senior managers, WHO staff, ward staff, pharmacists; recruit, designate, trained for leadership-task-shifted roles of hospital staff; assess for readiness and identified barriers, conducted needs assessment) 2. RESTRUCTURE: (revise profession roles—pharmacist became study coordinator and an additional pharmacist and medical student became trainers; change physical structure and equipment—supplied hand-rub to HCWs) 3. EDUCATE: (training on HH, distributed educational materials; made training dynamic—slide show, training film, and presentations) 4. FINANCIAL (fund and contract for clinical innovation—hand rub production) 5. QUALITY MGMT: (audit and provide feedback to HCWs)	1. FEASIBILITY: Reduction in HAI from baseline (18.7%) to follow-up (15.3%) (*p* = .453) observed using the WHO HH toolkit	1. FEASIBILITY: WHO HH improvement strategy was successfully implemented 2. COST: Economic production of alcohol hand rub was produced 3. PENETRATION: 224 HCWs were trained 4. ACCEPTIBILITY: HCW perceptions on some HH indicators improved (i.e. system change, education, providing feedback, etc.)
2	Brown et al. [50]	HIV prevention/safe conception practices associated with HIV prevention	1. EDUCATE: (Development of educational materials, HIV counseling guide, counseling messages to prevent HIV, brochures for HIV couples on safe conception practices and avoidance of HIV infection; Educational materials were distributed to providers and patients; Training was given to providers on all developed materials and on how to best counsel patients)	1. ACCEPTIBILITY: self- reported provider confidence in HIV/safer conception counseling and testing 2. FEASIBILITY: Providers stated that integrating counseling into HIV patient care was doable 3. SUSTAINABILITY: Increased confidence in HIV prevention/safe conception knowledge/practice sustained three weeks post-training (self-reported by providers)	1. PENETRATION: After counseling, patients (74%) were able to identify HIV treatment and viral suppression as effective strategies for safer contraception compared to pre-counseling (33%); Pre-training, only 10% of providers could identify the fertile period during a women’s menstrual cycle compared to 70% post-training; Pre training, only 66% of providers could identify safer contraception strategies to prevent HIV compared to 100% post-training; 116 potential participants screened to enroll in intervention—response rate for those who agreed to enroll was 42% for discordant couples, 34% for HIV+ women, and 100% for HCWs; No lost to follow-up among HCWs or patients 2. APPROPRIATENESS: self-report from HCWs that educational toolkit materials are culturally appropriate
3	Brown et al. [51]	Immunization	1. EDUCATE: (conduct training, make training dynamic: used PP, pictures, demonstrations, videos, and group discussions)	None	1. PENETRATION: 69 eligible HCWs could have participated in study; 69 did participate in study; 1 HCW was lost to follow-up; knowledge of HCWs increased immediately after the intervention, but then declined at 3 and 6 months; For the intervention group, overall knowledge scores increased significantly compared to non-intervention counterpart scores (*p* < .001) 2. FEASIBILITY: Factors identified as influencing HCW knowledge were assessed
4	Courtenay-Quirk et al. [52]	Post-exposure prophylaxis (PEP)	1. PLAN: (stakeholder buy-in and information sessions; visit different sites in the 3 countries; conducted an assessment of PEP barriers and BPE rates) 2. EDUCATE: (conducted training sessions on PEP; educational materials were distributed through the healthcare facilities)-posters, calendars, key chains 3. QUALITY MGMT: (update PEP operational plans)	1. FEASIBILITY: Formative research conducted at 9 health facilities prior to intervention to access potential challenges/barriers to PEP implementation; Factors hindering PEP were identified; BPE rates were high in HCWs, yet under-reported; PEP management not sufficient given low report of BPE incidences; Within the last 6 months, roughly 2073 (69%) of HCWs stated having a BPE. Of these HCWs who stated having a BPE, roughly 35.6% were not reported.	1. PENETRATION: (number of HCWs who attended training (n-2852)/compared to total HCWs who could have attended the training *N* = 4667) 2. APPROPRIATENESS: (HCW and healthcare management stated that tailoring each intervention to specific facility needs, HCW cadres needs, or messaging needs to be incorporated into intervention
5	Dahinten et al. [53]	ART (HIV therapy)	1. EDUCATE: (trained HCWs on “Pratt pouch”)	1. ADOPTION: (Pratt pouch was often used as a “bridge” until women could get to a healthcare facility, such that 73% of mothers used at least 3 pouches and 88% of mothers used less than 7 pouches; 2. FEASIBILITY: 90% of women who gave birth at home were able to use the Pratt pouch within three days of delivery 3. ACCEPTIBILITY: (26/30 mothers who gave birth at home stated that the pouch was easy to use or understanding the instructions of the pouch) 4. PENETRATION: Pratt pouch increased access to ARVs went from 35% to 94% (*p* < 05); 169 HIV+ pregnant women were surveyed. Of which, 160 enrolled in study)	1. PENETRATION: 41 HCWs from 8 different facilities were trained 2. SUSTAINABILITY: Three months after training, 8 nurses and 11 community-health workers were re-assessed and training knowledge was identified as retained
6	Durrheim et al. [54]	Surveillance/screening	1. PLAN: (stakeholder buy-in on surveillance system) 2. EDUCATE (develop effective educational materials (manual/training materials), trainings, and create a learning collaborative for nurses) 3. QUALITY MGMT: (audit and feedback-assessment for flaccid paralysis from hospital records, develop and organize quality monitoring systems for the surveillance system) 4. RESTRUCTURE: (Revise professional roles-nurses involved in active surveillance)	1. FIDELITY: During two year period, 14 cases of meningococcal disease occurred. All but one was notified and contained within 48-h period	1. SUSTAINABILITY: monthly meetings among nurses, networking, and feedback were identified as important mechanisms keeping the surveillance system on-going
7	Elnour et al. [39]	Waste management and proper disposal	1. EDUCATE: (training for HCWs on proper waste management; made training dynamic—used PP, group discussions, videos, demos, and health talks)	1. PENETRATION: Within the intervention group, self-reported practices (among those reporting good practice) rose from 42% to 55%. Similar increases in practice were self-reported for waste management practice indicators collected (ie safe waste separation)	None
8	Farley et al. [55]	MDR-TB treatment/monitoring	1. PLAN: (assessed for readiness via a SWOT analysis to identify strengths, weaknesses, opportunities, and threats in the current MDR-TB and HIV treatment model; Recruited, designated, and trained for leadership—one nurse case manager; Developed a formal implementation blueprint using the PRECEDE-PROCEED model; Conducted educational and ecological assessment, as well as, administrative and policy assessments for intervention 2. RESTRUCTURE: (revised professional roles via the introduction of nurse case manager role) 3. EDUCATE: (trained case manager on 6 proximal outcomes of interest associated with MDR-TB treatment management)	1. ADOPTION: 40 MDR-TB patients enrolled in the intervention and followed for the 6-month intervention period (aka to be followed by the nurse case manage 2. PENETRATION: 24% increased occurred between Cotrimoxazole preventative therapy at baseline to post-intervention; Active surveillance for adverse drug reactions increased by 75% from baseline to post-intervention.	1. PENETRATION: No lost to follow-up during 6-month intervention period of 40 patients; In terms of MDR-TB and HIV medical record concordance, nurse case manager identified 44% of the documented ART regimens were discordant between the medical records at baseline compared to post-intervention concordance, which was 100% between MDR-TB and HIV medical records
9	Fatti, G [56].	PMTCT (HIV therapy)	1. RESTRUCTURE: (revise professional roles via the use of a quality nurse mentor—whose responsibility is to build staff capacity and clinical management skills, ensure proper application of PMTCT guidelines) 2. QUALITY MGMT: (visit sites every two weeks and audit patient records with facility registers; address any data gaps, conduct re-fresher trainings for nurses if gaps exist)	1. PENETRATION: Estimated HIV testing in children increased 2-fold: 12.4% to 22.9% (*p* < 0001) Proportion of infant tested for HIV 6-weeks after birth increased: 68.7% to 76.7%(*p* < .0001); Repeat HIV testing at 32 weeks went from 38.5% to 46.4% (p < .0001); Zudovidine uptake increased from 80.9% to 88.1% (*p* < .0001); Of 27,458 pregnant women who could have been included in intervention, 4981 (18%) were included	1. PENETRATION: Nurse restructuring and quality management activities were introduced into 31 sites
10	Gous et al. [57]	Point-of-care testing (POCT)	1. EDUCATE: (senior level nurses trained how to use and evaluate POCT devices) 2. RESTRUCTURE: (senior level nurses at 2 clinic facilities are asked to task-shift duties to include POCT; new POCT devices are introduced into clinical system) 3. QUALITY MGMT: (POCT verification processes were undertaken through the study; Laboratory confirmation of POCT tests was also performed)	1. ACCEPTIBILITY: Nurses stated no difficulties in in performing POCT 2. FEASIBILITY: 70% of patients required 3+ POCT; On average, if CD4 counts were needed for the POCT, testing took roughly 1 h and 47 min; If CD4 was not needed, for 3 tests was 6 min; A total of 6% and 4.3% error rates for the POCT platforms were obtained in the two study sites	1. PENETRATION: 793 HIV+ patients were asked to enroll and 793 did 2. ACCEPTIBILITY: nurses stated a preference for quick reference charts as quick aids over longer training sessions 3. FEASIBILITY: POCT was implemented into 2 ART clinics; All POCT platforms passed verification; POCT did add to nurses’ already busy scope of practice
11	Holmen et al. [58]	Hand hygiene (hand washing)	1. PLAN: (stake holder buy-in established prior to intervention roll-out with hospital leadership; Ensuring procurement of alcohol hand rub both for HCWs and patient rooms in facility) 2. EDUCATE: (nurses and physicians attended HH training; posters and educational materials were placed in facility wards) 3. RESTRUCTURE: (providing HCWs personal alcohol hand rub) 4. QUALITY MGMT: (pre- intervention HH quality assessment via observations; audit and feedback given to facility administrators on HH compliance among HCWs post-intervention)	1. FIDELITY: Disparities among HH existed for both nurses and physicians pre-/post- intervention, with physicians being more compliant than nurses	1. PENETRATION: (9 out of 12 physicians and 54 out of 54 nurses attended the HH training: Knowledge increased among HCWs from 41.3% at baseline to 78.45(*p* < .001) post-intervention
12	Holmen et al. [33]	Hand hygiene (hand washing)	1. QUALITY MGMT: (purposeful re-examination of the HH intervention by conducting another post-intervention assessment via HH observation and interviews among nurses and physicians; performed audit/observation assessment of medical and nursing students in 2016)	1. SUSTAINABIITY: (Among all HCWs, HH compliance declined from the 2015 assessment to the current 2016 assessment from 68.9% to 36.8%(*p* < .001); Nurse-specific compliance decreased by 20.8%; physician compliance was reduced in 4/5 HH indications, nurse compliance reduced in 2/5; Similar to 2015, physician compliance was higher than nurse compliance, however, the difference had decreased by 9.7%; In 2016 assessment, there was no difference in HH compliance between medical or nursing students	1. FEASIBILITY: Production of alcohol hand gel that was provided to HCWs in 2015 assessment was no longer being made by the local pharmacist and distributed to HCWs
13	Howard et al. [59]	IPT (TB therapy)	1. PLAN: (engaged local government stakeholders before study to gain feedback and buy-in on intervention) 2. EDUCATE: (trained nurses on IPC protocols; distributed educational materials throughout clinics as reminders of IPC protocols) 3. RESTRUCTURE: (introduced new tools to capture patient data, such as a Family Care Enrollment form to screen family members for TB/HIV; Patients were reimbursed for clinic visits, provided mobile phones, airtime, and sent reminder SMS messages to them) 4. QUALITY MGMT: (tools were developed for intervention monitoring)	1. ADOPTION: IPT initiation rates 2. PENETRATION: IPT completion rates; IPT adherence rates; ART adherence rates; changes in CD4 counts; retention in HIV care	1. ACCEPTABILITY: Majority of patients and HCWs stated that IPT combination intervention was agreeable
14	Imani et al. [60]	Screening, diagnosis, therapy	1. EDUCATE: (trained HCWs on childhood infectious diseases: TB, HIV, malaria, and others, specifically on the diagnosis and therapy components of these diseases 2. QUALITY MGMT: (on-site supervision was provided for 9-months to HCWs, whereby mentorship and extra training were provided)	1. FEASIBILITY: Relative risk ratios comparing intervention to control relative risk showed no difference in improvements in screening, diagnosis, and therapy initiation	None
15	Jere et al. [61]	Universal precautions AND HIV prevention associated with universal precautions	1. EDUCATE: (provided training for HCWs on universal precautions and HIV preventions; trainings were made dynamic in that they incorporated rehearsal of key skills with feedback) 2. QUALITY MGMT: (developed tools for intervention monitoring, which included pre-/post- assessments and observations	None	1. FEASIBILITY: At baseline, overall universal precautions knowledge was higher in the control group
16	Jones et al. [62]	Standard Precautions/UP	1. PLAN: (a collaboration of stake- holders was established between the MOH and the Global Health Alliance of Western Australia (GHAWA) to better understand why clinical practice had not changed among HCWs after an infectious disease training; An IPC needs assessment was performed by GHAWA; conducted healthcare facility visits to gain a sense of IPC challenges and cultural contexts) 2. EDUCATE: (developed an IPC course to fill in some of the gaps from previous courses/trainings; received stakeholder feedback and modified the course based on local context; training was made dynamic via role-plays and Glitterbug!)	1. FEASIBILITY: Health facilities lacked running water, lack of resources, storage for IPC materials	1. ACCEPTABILITY: HCWs reported they thought the training was useful and informative
17	Jones-Konneh et al. [63]	Standard precautions and transmission- based precautions for EVD prevention	1. PLAN: (a collaboration of stake-holders was established to be able to cover all training needs, partners included: IOM, COMHAS, MOH, and RSLAF 2. EDUCATE: (HCWs were trained on the relevant standard precautions and transmission-based precautions to provide patient care and to maintain their own safety; Training was made dynamic—mock ETU were used, skills stations, clinical cases, and lectures)	None	1. PENETRATION: 6206 HCWs were trained 2. FEASIBILITY: Anxiety associated with providing care to EVD patients decreased after training
18	Kaponda et al. [64]	Universal precautions AND HIV prevention associated with universal precautions	1. EDUCATE: (provided training for HCWs on universal precautions and HIV preventions; trainings were made dynamic in that they incorporated rehearsal of key skills with feedback) 2. QUALITY MGMT: (developed tools for intervention monitoring, which included pre-/post- assessments and observations	None	1. ACCEPTABILITY: HIV patients were asked about if HCWs had discussed any of the training materials with them AND how they felt about the material delivered; At baseline, only 28% of HCWs had discussed HIV prevention with patients compared to37% post-intervention(*p* < .01)
19	Karari et al. [65]	ART (HIV therapy)	1. PLAN: (established an academic partnership to initiate Uliza; a publicity meeting was conducted introducing Uliza to HCWs) 2. EDUCATE: (promoting Uliza vai educational sessions to HCWs) 3. QUALITY MGMT: (reminded HCWs of Uliza via text messages; introduced Uliza: telephone consultation service to improve HIV care/ART; tools/surveys developed to evaluate the implementation of Uliza; chart audits at healthcare facilities were reviewed to assess if Uliza advice was actually implemented by HCWs)	None	1. PENETRATION: 296 calls from 79 different HCWs used Uliza within the first year of its implementation; 58.4% of HCWs made 2+ calls; 69% of calls came from district hospitals or healthcare centers 2. ACCEPTABILITY: Among users of Uliza, all most all (94+%) agreed that the service helped them with providing better patient care, met their expectations, convenient, and timely. 3. FEASIBILITY: two important barriers to implementation success: cell phone coverage in certain rural areas, and delayed response from Uliza consultants; Nonusers stated they did not use the service because they did not know about it, did not have questions, or used other resource materials available to them
20	Kerrigan et al. [66]	TB screening/active case finding	1. PLAN: (Focus group discussions and in-depth interviews with key stakeholders (HCWs, patients, and family members) was performed to identify which active case finding method for TB (clinic-based, home-based, or incentive-based) would be the best option for a future trial)	None	1. ACCEPTABILITY: All study participants stated the all three strategies would be acceptable, yet each method had its pros and cons, and some methods may be better targeted to specific patient populations. For example, patients stated clinic-based methods were generally acceptable and not out of the ordinary, however, they want to ensure patients were not being stigmatized. Thus, they suggested that ALL patients (not just some patients, like HIV+) be screened; HCWs stated they favored incentive-based system best 2. FEASIBILITY: Clinic costs and transportation times were also listed as issues that might inhibit this strategy’s successful implementation; Some TB patients stated they liked the home-based method better, as it allowed patients to feel more comfortable, less costs, but it did have the potential to create stigma; In terms of the material-based method, many participants stated food and money were good incentives, yet they questioned if the government would be able to implement this form of incentive system. Additional concerns related to this method were around if this method was sustainable.
21	Kunzmann et al. [67]	“Best Care Always:” an evidence- based HCAI prevention bundle	1. PLAN: (consensus from local stakeholders agreed that a VAP bundle needed to be implemented on PICUs) 2. RESTRUCTURE: (task-shifting via the creation of a “VAP champion” role for nurses; nurse teams of 5 members were created to implement the VAP bundle; doctors required to complete VAP identification screening form; VAP Coordinator position was created) 3. EDUCATE: (all staff involved in the implementation of the VAP bundle were trained on the bundle; one-to-one teaching sessions occurred between VAP Coordinator and nursing staff; educational materials were made available throughout the PICU) 4. QUALITY MGMT: (new tool adapted to screen for VAP; regularly VAP monitoring for VAP introduced)	None	1. ACCEPTABILITY; Full-time VAP Coordinator, whose duties would not be clinical, was well received by healthcare facility administration (this newly created position would not cause senior nursing staff to be pulled away from the bedside) 2. FEASIBILITY: Initial implementation of VAP bundle was not successful; Resources shortages, limited number of nurses all contributed to implementation challenges; Upon initial implementation, many challenges arose that required changing the implementation approach of the VAP bundle intervention. For example, within first 4 months, data collection was unreliable, compliance low, 5-member nurse team had little time to teach and monitor staff on bundle, resistance from nurse staff in wanting to implement the VAP bundle, and PICU team buy-in was challenging-no sense of urgency to change 3. SUSTAINABILITY: After the VAP Coordinator position was eliminated, the intervention continued for 3 months
22	Labhardt et al. [68]	PMTCT (HIV therapy)	1. RESTRUCTURE: (HIV kits, pocket guides for HIV ART distributed to 70 health care facilities) 2. QUALITY MGMT: (Throughout study, HIV kit inventories were captured; staff knowledge was assessed on ART; Senior nurses developed an inventory tracking tool for ART resources at the facility level) 3. EDUCATE: (HCWs were provided HIV/ART training, training was made dynamic via the use of lectures, demonstrations, teach-backs, interactive plenary sessions; on-site supervision occurred after training as well)	None	1. PENETRATION: HIV materials to deliver ART was distributed to 70 facilities; (44 healthcare facilities (63%) contained full equipment for HIV testing; 16 (23%) had stocked PMTCT drugs; Only 14 (20%) had both 2. FEASIBILITY: Physicians confirmed that PMTCT drugs had reached district-level facilities; yet materials and funds for training had not
23	Levy et al. [69]	Standard precautions and transmission- based precautions for EVD prevention	1. PLAN: (coalition established between MOH and CDC, NGOs; some partners procured PPE, others organized logistics associated with training in the district PHUs) 2. EDUCATE: (IPC curriculum, training materials, and health promotion materials were produced; on-site PHU training occurred for all staff; Train-the-trainer strategy used to educate staff in 1200 PHUs nationwide) 3. COST: (funding for intervention strategies was provided from a variety of international, external sources) 4. QUALITY MGMT: (district teams performed initial PHU assessments, made recommendations, and returned a week later to perform quantitative assessment of the PHU; Additional feedback and training was provided at this time)	None	1. PENETRATION: 4264 HCWs trained in 14 districts; over 94% of PHUS received training/PPE supplies
24	Lewin et al. [70]	TB therapy	1. EDUCATE: (training materials produced that incorporated a lot of staff self-reflection on TB care, addressing barriers to care, and empowering staff to implement system changes; training on the newly produced materials was carried out)	1. PENETRATION: rates for successful completion of treatment improved more in the intervention clinics than in the control clinics, yet these differences were not statistically significant)	1. FEASIBILITY: Training was successfully conducted in all clinics, except for 1; complete pre-and post-intervention data were obtained for all clinics, except 1; all clinic-based records matched 100% of the laboratory records 2. ACCEPTIBILITY: Clinic staff stated that they generally approved and liked the intervention
25	Liautaud et al. [71]	HIV/TB IPC	1. FIANANCE: (University of Free State received funding for this program from Canada’s Global Health Research Initiative) 2. EDUCATE: (HCWs were trained on HIV/TB IPC; training was made dynamic—via the use of collaborative projects that had to address a specific HIV/TB IPC challenge at the HCWs place of work; HCWs had to develop proposals, initiate research models, and collect data)	1. FEASIBILITY: Barriers to intervention implementation were identified: not enough time, lack of resources, logistical challenges, and institutional capacity; Lack of computer skills to develop HIV/TB IPC materials for research/data collection was a barrier to many HCWs	1. ACCEPTIBILITY: HCWs stated that this intervention program was good, eye-opening, and substantial; HCWs felt they had learned a lot about research that they previously had no exposure to
26	Liu et al. [72]	Ebola ETU safe design/layout for patient isolation and infection reduction; standard precautions; transmission-based precautions	1. RESTRUCTURE: (facilities to care for EVD patients was completely physically re-designed to adhere to IPC guidelines; cameras install into EVD units for close patient monitoring; Initiating a buddy-system for EVD patient care) 2. EDUCATE: (new EVD guidelines were developed; new tools for donning/doffing were produced—41 step PPE wearing guide was developed; training for EVD IPC provided to all HCWs working with EVD patients) 3. QUALITY MGMT: (strict supervision and inspection of all staff’s PPE prior to ETU entry was required; site inspections occurred regularly with video surveillance; Feedback was immediately provided to HCWs if PPE errors were observed)	None	1. PENETRATION: 1520 individuals were trained in EVD IPC; 80 local HCWs were trained in EVD IPC 2. FEASIBILITY: EVD facilities were constructed
27	Mahomed et al. [73]	IPC Practices (including standard precautions and transmission-based precautions)	1. RESTRUCTURE: (trained nurses were used to fill out ICATs for given IPC assessment areas, like hand hygiene or isolation/quarantine) 2. EDUCATE: (nurses were trained on the ICAT tool) 3. QUALITY MGMT: (introduction of the ICAT tool to assess how well IPC is being implemented in ICUS)	None	1. PENETRATION: IPC practices associated with study were carried out in 6 public and 5 private ICUs 2. FEASIBILITY: Nurses successfully completed ICAT assessments
28	Miceli et al. [74]	HIV prevention/ART, TB screening/therapy, malaria screening/therapy	1. EDUCATE: (implemented the Integrated Management of Infectious Disease training program; made training dynamic via case studies, group discussions, and small group work; On-site support and continuing education for HCWs was provided throughout 9-month study period) 2. QUALITY MGMT: (performed observations of HCWs practice for a total of 20 observations; assessed site performance via a surveillance system; assessment of population outcomes)	None	1. PENETRATION: 72 HCWs trained on IMID; HCWs applied complex clinical reasoning concepts by: analyzing 40–50 cases, discussing 20–30 presentations from their peers, 36 h of clinical placement, and discussing with 20 physicians
29	Mbombo & Bimerew [75]	PMTCT (HIV therapy)	1. EDUCATE: (trainings performed in both midwifery and PMTCT for nursing students: training was dynamic via skills lab, visualization processes, guided practice, and independent practice)	1. PENETRATION: Of 154 students, 107 (69.5%) provided intrapartum ARV prophylaxis to pregnant women; Of 116 students, 75.3% conducted neonatal ARV prophylaxis, (23 or 14.5%) performed 15 neonatal ARV prophylaxis procedures	1. PENETRATION: 134 students performed the HIV pre-testing counseling competency (only 119 or 77.3%) completed the required 10 pre-test cases; 132 students completed the post-test counseling competency (only 115 or 74.7%) completed the required 10 post-test cases; Of the 144 student who submitted their competencies, 135 (87.7%) performed the required 7 rapid fingersticks for HIV testing; 121 (78%) of students provided dual ARV therapy (74 or 48.1%) competed the required 10 dual therapy sessions;
30	Ogoina et al. [76]	Standard precautions and transmission-based precautions for EVD prevention	1. PLAN: (established partnerships between the Niger Delta University Teaching Hospital (NDUTH) and the Bayelsa State Ebola Task Force, the MOH, and international partners, like WHO; appraisal of the hospital’s EVD preparedness was assessed via the WHO Ebola checklist) 2. RESTRUTURE: (EVD response team was created at hospital; Facility altered to include isolation/quarantine space) 3. EDUCATE: (sensitization EVD workshops were provided to all HCWs) 4. FINANCIAL (funds from the state allowed for an isolation ward to be designed and built) 5. QUALITY MGMT: (assessments performed to assess HCW EVD knowledge, fears, myths; continuous IPC evaluations were performed throughout epidemic	1. PENETRATION: 3 EVD “alarms” were reported, which turned out to be false/non-EVD cases 2. SUSTAINABILITY: a significant outcome of this study is associated with sustainability. Once the EVD outbreak was over in Nigeria, the IPC activities were not sustained	1. PENETRATION: Among 500 HCWs, 189 completed the survey on EVD; 3-false alarms of EVD were reported 2. FEASIBILITY: Some HCWs were reluctant to be a part of the EVD team—some asked for stipends and life insurance to participate in EVD team; many HCWs refused to work in an isolation ward; Among 189 HCWs, 82% believed the misconception that EVD can be prevented by avoiding crowds; 56% of HCWs believed the misconception that patients with a fever should be treated like an EVD case; Hand gloves, sanitizers, and hand soap quickly ran out upon initiation of the intervention 3. ADOPTION: an isolation ward was established; hospital procured PPE, alcohol hand gel, established the use of an incinerator
31	Otu et al. [77]	Standard precautions and transmission-based precautions for EVD prevention	1. PLAN: (partnerships between the national government, cell phone companies, and other partners were established) 2. EDUCATE: (HCWs were provided an Ebola Awareness Tutorial (EAT); EAT was designed and developed by Information Control Technology and IPC experts) 3. QUALITY MGMT: (Pilot testing of EAT to assess diagnostic and management responses to EVD; pre- and post-intervention assessments performed to evaluate EAT)	1. ACCEPTIBILITY: Positive response to using PPE to prevent the spread of EVD.	None
32	Parker et al. [78]	Safe Water System (Hand hygiene)	1. PLAN: (partnerships between the CDC, CARE International, PSI, and the Maternal and Child Health Clinic in Homa Bay) 2. EDUCATE: (training in SWS and proper hand washing technique were provided to all nurses; educational materials post training were also provided nurses)	None	1. PENETRATION: All 11 nurses received the SWS and hand-washing training; Post-training, 7 nurses scored 100% of the post-test assessment, 3 scored 91%, and 1 scored 62%; 10(91%) of nurses reported using hand hygiene and SWS materials to teach clients using materials from the trainings they received
33	Richards et al. [79]	“Best Care Always:” an evidence- based HCAI prevention bundle	1. PLAN: (coalition established between private hospital groups (Netcare), healthcare professionals, and the National Department of Health; ICU staff were familiarized with the bundle) 2. RESTRUCTURE: (CLABSI bundle implemented; On-going monitoring was integrated into ICU nurses’ roles and responsibilities) 3. EDUCATE: (multiple regional learning sessions about BCA campaign and CLABSI bundle were provided for nurses; guidelines were given to nurses; educational materials were made available) 4. FINANCE: (BCA campaign implemented using funds from private hospital nursing budgets or the overall hospital budget) 5. QUALITY MGMT: (Central-line bundle checklist were implemented for intervention monitoring; audits were regularly conducted; staff kept engaged via regular audit and surveillance data provided in visual forms, like run charts and team meetings	None	1. FEASIBILITY: CLABSI bundle successfully implemented in 43 healthcare facilities; Overall study recorded 1,119,558 central-line-days
34	Samuel et al. [80]	Hand hygiene (hand washing)	1. PLAN: (strong support and buy-in form government regarding intervention; multi-disciplinary infection prevention committee was initiated) 2. QUALITY MGMT: (feedback from healthcare workers and patients obtained regarding hand washing practices; direct hand washing observations were carried out to ensure quality practice: data triangulation and quality checks were performed; immediate feedback was given and correct hand washing practice demonstrated whenever requested during weekly facilitation and couching visits; Post-intervention workshops were carried out to engage hospital management team and HCWs in problem solving sessions) 3. EDUCATE: (In-service training on hand washing was provided to all HCWs; Facilitation and coaching visits were made weekly) 4. RESTRUCTURE: (HCWs provided hand towels and soap; portable water tap for hand washing was placed on wards)	None	1. FEASIBILITY Lack of resources (soap and towels) stated by HCWs; soap, towels, and water taps were made available to HCWs on the wards; Observations conducted assessed that all wards had running water, 60% had functioning and conveniently located sinks)
35	Schmitz et al. [81]	Hand hygiene/washing	1. PLAN: Hospital leadership was involved in the design, conception, and implementation of project; Baseline evaluations/observation were conducted 2. RESTRUCTURE: Alcohol, soap, and personalized bottles of hand gel were made available to HCWs; New roles were established called “Hand Hygiene Champions, who were hospital leaders who supported HH changes at the facility) 3. EDUCATE: HCWs were trained using WHO HH strategies; Posters and visual aids were put around the facility; Informal teaching sessions were provided HCWs during daily rounding) 4. QUALITY MGMT: Monitoring and feedback on HH practices was given to HCWs; A post-intervention evaluation was conducted; HCWs were asked to complete a survey focused on HCW acceptance of the WHO campaign	None	1. FEASIBILITY All 11 patient wards at hospital had functioning sinks with a 1:4.6 patient/bed ratio; Only 20% of the sinks had soap 2. PENETRATION: 212 HCWs were approached to complete the post-intervention survey, and 161 HCWs completed; Among the HCWs who attended the training, 85.4% (111/130) and 80.8% (104/130) stated that the training increased their knowledge of HH and impacted their practice respectively 3. ACCEPTIBILITY: 64.0% of HCWs stated that they preferred hand gel to soap, and hand gel would improve their HH practice
36	Shumbusho et al. [82]	ART (HIV therapy)	1. PLAN: (partnership established between MOH, FHI (NGO), and 3 health center nurse staff) 2. EDUCATE: (nurses were trained by an FHI physician; on-going physician support with weekly supervision and mentoring at each PHC) 3. RESTRUCTURE: (ART management tools were modified for nurse use during task-shifted roles) 4. QUALITY MGMT: (created checklist were created to aid nurses to collect and assess patient data)	1. PENETRATION: Of the 1076 patient enrolled in the study, 435 (40)% were initiated on ART by nurses; CD4 counts pre-ART to 6-month follow-up changed from 97 to 128 cells/ul; CD4 count from 6-months to 24-months changed from 79 to 129 cells; Mean weight gained within the first 6 months of ART initiation was 1.8 to 4.3 kg; In the last 6 months, mean weight increases were 0.5 to 1.7 kg	1. PENETRATION: 3 nurses received ART training 2. FEASIBILITY: Task-shifting successfully implemented
37	Speare et al. [83]	Surveillance/screening	1. PLAN: (collaboration established between RSA provincial government, RSA universities, and Australia) 2. RESTRUCTURE: (RSA provincial government established communicable district control coordinator (CDCC) 3. EDUCATE: (nurses were trained to conduct epidemiological field surveys)	None	1. PENETATION: 16 out of 20 nurses were trained in the CDCC curriculum; 2 nurses were able to present research they conducted while in their CDCC role at a conference; some nurses also co-authored manuscripts 2. ACCEPTIBILITY: 15 of the 20 nurses stated the training materials/sessions were appropriate for their needs
38	Tillerkeratne et al. [84]	CAUTI Infection Prevention and Control	1. PLAN: (pre-intervention surveillance was performed to obtain baseline data on CAUTI rates and practices) 2. EDUCATE: (training was performed for HCWs to address correct catheter placement/management; Reminder signs were placed over patient beds to act as visual HCW reminders; Training was made dynamic via videos, lectures, demonstrations; Discussions had on HH, sterile gloves, and antisepsis) 3. QUALITY MGMT: (Nurse matrons performed weekly infection prevention rounds on catheterized patients to assess if catheters were still needed for said patients; Post-intervention, surveillance conducted to assess CAUTI rates)	1. FEASIBILITY: Limited hospital supplies reported: bed-pans/urinals stated as reasons for catheter-placement during pre-intervention phase of study	1. PENETRATION: 125 patients received catheters during this study; 82 in the pre-intervention phase AND 43 in the post-intervention phase
39	Uneke et al. [34]	Hand hygiene/washing	1. PLAN: (consultations/advocacy meetings were conducted between the Research team and stakeholders: Chief Resident Doctors and Heads of Nursing Services) before study commencement 2. EDUCATE: (training sessions on HH were conducted; HH reminders were placed within the wards/hospital) 3. RESTRUCTURE: (Alcohol-based hand gel was made available on all the wards) 4. QUALITY MGMT: (pre- and post-intervention observations were performed to assess for HH compliance)	1. FEASIBILITY: Inadequate water supply, limited access to soap and towels, lack of awareness of HH, insufficient number of HCWs, and absent guidelines for HH were all listed as reasons for limited HH compliance)	1. PENETRATION: 202 HCWs were trained
40	Uneke et al. [85]	Disinfection (of medical equipment)	1. PLAN: (pre-intervention assessments were performed to assess HCWs KAP of stethoscope handling and maintenance; Microbiological assessment were performed on HCW’s stethoscopes; Baseline compliance of stethoscope disinfection practices of HCWs was performed; FGD were conducted to assess factors with limited stethoscope compliance among HCWs) 2. EDUCATE: (training In workshops on stethoscope disinfection practices were provided to HCWs) 3. RESTRUCTURE: (Alcohol-based hand gel was procured and placed on wards) 4. QUALITY MGMT: (post-assessments performed on stethoscope disinfection practices and microbiological assessments)	1. FEASIBILITY: Reasons by HCWs for non- compliance with stethoscope disinfection were provided) 2. PENETRATION: Pre-intervention assessments revealed that no doctors regularly disinfected their stethoscopes after seeing patients, yet 39.2% of nurses did; Post-intervention, 89 stethoscopes were microbiologically tested for bacteria, roughly 20.2% of them were contaminated with bacterial agents; Following the intervention, stethoscope contamination was reduced by 58.3% compared to pre-intervention rates; Post-intervention, 100% of HCWs cleaned their stethoscopes after each patient	1. PENETRATION: 202 HCWs were trained in a series of workshops; Post-intervention, 89 HCWs were asked to provide their stethoscopes for microbiological assessment, and 100% of them did
41	Van Rie et al. [86]	HIV Counseling/Testing AND TB Prophylaxis	1. EDUCATE: (nurses received HIV counseling and testing training for 2 weeks) 2. RESCTRUCTURE: (HIV counseling and testing services were provided to patients utilizing three different delivery models: referral to a free-standing VCT clinic, Referral to counseling/testing center to which the TB clinic belongs, or TB nurse offered to patient and provide HIV counseling/testing	1. PENETRATION: Cotrimoxazole prophylaxis was initiated in 89.9% of HIV co-infected patients with TB 3. SUSTAINABILITY: Of those who initiated the CTX at the TB clinic, 88.4% were still on it at the end of 8-months of TB treatment or until time of death.	1. ACCEPTIBILITY: The proportion of patients accepting HIV counseling/testing was significantly lower (68.5%) at the clinic that referred patients to a free-standing VCT center compared to 94.8% acceptance rate at the clinic with on-site referral and 97.7% at the clinic where counseling/testing was conducted by a TB nurse (*p* < 001) 2. ADOPTION: 3 TB clinics implemented the intervention 3.FEASIBILITY: 1238 (99.4%) of the 1246 patients registered at the 3 TB clinics had available data 4. PENETRATION: 10 nurses trained on HIV counseling/testing and TB prophylaxis
42	Wanyu et al. [87]	PMTCT (HIV therapy)	1. PLAN: (Study supervisory staff met with village health committee and educated them about HIV and PMTCT; If committee agreed to participate in study, local trained birth attendants would be sent for additional training) 2. EDUCATE: (Trained birth attendants were further trained in PMTCT protocols, including HIV counseling, peripartum administration of Nevirapine to mother and baby, and HIV testing)	1. PENETRATION: Trained birth attendants counseled 2331 women; Of the 2331 women counseling, 2310 (99.1%) agreed to initial HIV testing using OraQuick; Of the 42 women who delivered at the primary health centers, 37 (88.1%) received Nevarapine prophylaxis is; Of the 42 newborns delivered by trained birth attendants, 36 (85.7%) were treated with Nevarapine after birth; Of the children whose mother’s had a positive Ora Quick test, 14 were tested for HIV at 15 months)	1. PENETRATION: 30 trained birth attendants agreed and received training
43	Welty et al. [88]	PMTCT (HIV therapy)	1. EDUCATE: (Nurses and trained birth attendants were trained how to conduct HIV counseling/testing and provide Nevarapine prophylaxis) 2. COST: (A variety of stakeholders provided financial support for this intervention, such as the Elizabeth Glaser Pediatric AIDS Foundation, Axios Foundation, Boehringer Ingleheim Pharmaceutical Company	1. PENETRATION: 68,635 women received pre-test counseling; 63,094 (91.9%) of women were screened for HIV; Of those screened for HIV, 5550 (8.7%) were HIV positive; Of those who were HIV positive, 5433 (97%) were provided Nevarapine	1. PENETRATION: 690 nurses, nurse aids, and trained birth attendants were trained to provide PMTCT in 115 facilities in 6 out of ten provinces in Cameroon
44	White et al. [89]	IPC Practices during surgeries (disinfection of equipment, decontamination of equipment, disinfection of environment)	1. EDUCATE: (3 different training methods were used to promote the use of the WHO Surgical checklist—nurses were only trained in one method (the team method: an intra-professional training modality) 2. QUALITY MGMT: (Training effectiveness was measured post-training once HCWs returned back to their home healthcare facilities. Only 3 out of the 4 WHO areas of patient care were assessed—Measurement of Surgical services was not conducted)	1. APPROPRIATE: Upon returning to their home healthcare facilities, all HCWs who trained on board or in the team modalities, but none who trained only in the classroom, stated the checklist improved infection control 2. ADOPTION: HCWs stated they felt improvement in decontamination and washing of surgical instruments, using bleach solutions, not picking instruments off the floor, and cleaning up blood as soon as it hits the floor)	1. ACCEPTIBILITY: For those HCWs in the “team modality,” they all felt that these way of training was good and improved infection control in their hospitals 2. FEASIBILITY: Implementing the checklist in it’s entirety was not achieved
45	Xi et al. [90]	PPE use	1. PLAN: (To prevent Ebola infection in HCWs, video cameras were installed in the ETU to perform surveillance of HCWs as they doffed their PPE) 2. EDCUATE: (nurses were trained to supervise HCWs as they doffed their PPE via video surveillance to ensure that each step of doffing process was successfully performed by HCWs) 3. QUALITY MGMT: (Nurses monitored HCWs during the doffing process; gave real-time feedback during the process of mistakes were made via a communication system installed in the ETU; Nurses would also record any mistakes and discuss these with HCWs after doffing; Nurses made a standardize table of all of the required doffing steps to be used during surveillance activities)	1. FIDELITY: 1797 inappropriate doffing actions were identified and corrected; In the first week, the error rates for each doffing step was between 0.60% to 50.60%; In the second, third, fourth, and fifth weeks, the error rates were 0–19.05%, 0–0.89%, 0–1.19%, and 0–0.89% respectively.	1. PENETRATION: 8 nurses were trained in required PPE doffing procedures; A total of 1680 counts of doffing PPE were recorded
46	Zaeh et al. [91]	TB screening/IPT Prophylaxis	1. PLAN: (pre-intervention assessments were performed to collect baseline data see if HIV+ patients at the health facility had been screen for TB and/or been put on IPT) 2. EDUCATE: (HCWs were trained on TB screening and IPT; Reminder posters were posted throughout the HIV clinic) 3. QUALITY MGMT: (The WHO TB screening checklist was added to each patients’ chart to help HCWs remember to complete it; Post-intervention assessments were conducted to address any change after the intervention)	1. PENETRATION: 751 HIV+ patients were evaluated during the study; Post-intervention, 94% of HIV+ patients were screening for TB compared to only 22% pre-intervention; Nurses originally screened 3% of patients, yet after the intervention, they screened 100% (*p* < 001); Among the patients with a negative symptom screen who were eligible for IPT, 81% were put on IPT compared to only 4% (*p* < 001) pre-intervention; Nurses initiated 90% of HIV+ patients on IPT post-intervention compared to only 17% (*p* < 001) pre-intervention)	1. PENETRATION: 2 nurses trained in TB screening/ IPT
47	Bedelu et al. [92]	ART (HIV therapy)	1. PLAN: (Partnership established with MSF to deliver ART using a task-shifting approach and de-centralized HIV care) 2. EDUCATE: (Nurses and CHWs were provided training on HIV management, including ART, PMTCT, and TB diagnosis) 3. RESTRUCTURE: (Task-shifting was utilized to have HIV ART be nurse-initiated instead of physician-initiated) 4. QUALITY MGMT: (All clinics with task-shifted nurses received support from physicians via a mobile visit; Intervention evaluation was conducted by a quality control team (consisting of one physician and one nurse) on a quarterly basis)	1. PENETRATION Post-intervention, 2200 people were receiving ART; Pre-intervention, 50% of service users at the hospital and 40% at the clinics arrived with CD4 counts less than 50 cells/mm. Post-intervention, the number of patients with CD4 counts less than 50 cells/mm had decreased to 16& at both facility types 2. SUSTAINABILITY: Cohort analysis of people who received ART treatment for longer than 12 months showed satisfactory immunological recovery and viral suppression;	1. FEASIBILITY: Closer proximity and acceptability of services at the clinic level (compared to hospital level) led to faster enrollment of people on ART and better patient retention 2. PENETRATION: Only 2% of people were lost to follow up
48	Elden et al. [38]	TB screening/intensive case finding	1. EDUCATE: (nurses and HIV counselors attended a 2-day training course on TB screening tool and intensive case finding) 2. RESTRUCTURE: (Transportation systems were introduced to allow for the more effective delivery of sputum samples; One full time nurse and one TB/HIV Coordinator were hired to coordinate intervention) 3. QUALITY MGMT: (TB intensive case finding screening tool was developed and used to monitor intervention progress; Monthly supervisory visits were made to the hospital and clinics to ensure use of the new ICP tool; an MEQ system was implemented to assess patient treatment initiation if TB positive) 4. COST: (Nurse coordinator, outreach coordinator, and TB coordinator all received cellular phones with monthly credit; Clinics were given month cellular telephone credit to call hospitals)	1. PENETRATION: During 3 month study period, 1467 HIV+ patients were screened for TB (1129 from hospital; 338 from clinics); Of those screened, 365 (25%) were identified at TB suspects; Using 1467 as the denominator, 28 (2%) of HIV+ patients were identified as TB positive	1. ADOPTION: Proportion of HIV+ patients screened was higher in the clinics than in the hospital—potentially due to staff motivation or rigorous application of the screening tool (*p* < 001); 53% of patient did not return their TB specimens for testing—potentially due to financial and geographical barriers, difficulty producing sputum, and patients not prioritizing testing) 2. FEASIBILITY: Overall, ICP was implemented into the hospital and clinics successfully)
49	Charalambous et al. [93]	ART (HIV therapy) TB screening/INH therapy	1. PLAN: (Stakeholder buy-in and informational sessions were conducted with primary health service nurses, mine management, union representatives to address any potential concerns/issues with the introduction of the new HIV clinic on-site at the mines) 2. RESTRUCTURE: (a new HIV clinic was proposed to be located and operationalize for HIV+ miners; Staff were recruited to service the new HIV clinic, including a professional nurse and two enrolled nurses) 3. EDUCATE: (All staff involved in patient care at the HIV clinic received training in HIV management (ART) and TB screening and prophylaxis) 4. QUALITY MGMT: (Regular meetings were held with nurses to discuss issues concerning monitoring and supply of preventative therapy; A liaison nurses visited the primary care nurses to monitor record keeping and to provide additional education to staff about preventative therapy)	1. PENETRAION: Of the 1773 new HIV clinic attendees, 48 (3.7%) were found to have TB upon screening; 1190 (67%) of clinic attendees were eligible for INH, 966 (82%) initiated INH.	1. PENETRATION: 1773 patients attended the new HIV clinic; Of the 1773 patients who attended the clinic, 1270 (72%) were still active attendees over a 29-month period; This framework was used to establish two other similar clinics at mining companies in RSA 2. ACCEPTIBILITY: Patients attending the clinic had positive feelings about the preventative therapy and were confident about the benefits of attending the clinic; Clinic attendees were very enthusiastic about ART initiation 3. FEASIBILITY: new HIV clinic was established at mining facility for miners
50	Fairall et al. [40]	ART (HIV therapy)	1. EDUCATE: (Nurses received HIV training sessions about prescribing ART and potential side effects, as well as, the PALSA PLUS guidelines, which provide care for respiratory disorders, like TB; Nurse managers would provide additional educational sessions to intervention nurses) 2. QUALITY MGMT: (24 physicians would mentor and support nurses initiating ART to patients at the intervention clinics) 3. RESTRUCTURE: (Physician roles were task-shifted to nurses to initiate ART for patients)	1. ADOPTION: (All 16 clinics were able to implement phase 2, re-initiating ART in patients who are already taking it	1. PENETRATION: 103 nurses trained in ART; All 16 intervention clinics were able to successfully implement 2 out 3 phases of the intervention 2. FEASIBILITY: Task-shifting ART to nurses in a large-scale public sector program did not improve survival of patient not yet taking ART with CD4 counts of 350 cell/mm or less, but it did in patients with CD4 counts of 201–350 cell/mm, although the difference was not significant; 2 clinics could not implement phase 3 due to difficulties with staff and drug distribution
51	Harrison et al. [94]	STD Syndromic case management	1. EDUCATE: (Nurses received STD case management training, including STD drugs, counseling, condom protection, contact tracing, and syndrome packets that have STD specific treatment, condoms, partner cards, and patient information leaflet; Nurses also participated in problem-solving exercises to define objectives to improve the quality of STD management) 2. QUALITY MGMT: (Three follow- up sessions were held at each intervention clinic; monthly follow-up visits to the clinic by the district STD team provided continued STD management support) 3. RESTRUCTURE: (Nurses were asked to implement the use of syndrome packets to patients; Simulated patients were used to assess training. Simulated patients were come to the intervention clinics and use a standard script, presenting as a patient with an STD)	None	1. PENETRATION: 5 nurses were trained on STD management and syndrome packets; Post-intervention, the intervention is now being implemented within the 5 control clinics 2. ACCEPTABILITY: syndrome packets were well received by patients and nurses—they are now the standard of care in the intervention clinics 3. FEASIBILITY: Program was demonstrated by its integration within primary healthcare services
52	Naidoo et al. [95]	TB diagnosis, treatment, and treatment monitoring	1. EDUCATE: (HCWs were trained in TB diagnosis, treatment, DOTS, drug management, etc.)	None	1. PENETRATION: Of the 818 HCWs who were invited to participate in the training, 585 (71.0%) participated in at least part of the training program; Of the 818 HCWs, 267 (46%) attended the training and completed both the pre- and post- training knowledge assessments; For the 267 HCWs, percentage of correct answers on assessments rose from 59.9% pre- training to 66.5% post-training; Nurses had the lowest knowledge scores post- training—their scores were the lowest in TB patient management and TB program monitoring; Though nurses scored the lowest on the assessments, their improvements post training compared to pre-training were significant (*p* < 001)
53	Morris et al. [96]	ART (HIV therapy)	1. EDUCATE: (Clinical officers were trained to prescribe ART, manage toxicities and opportunistic infections; Nurses were trained in triage, which ensured that all assessed patients were prioritized for care based on clinical need) 2. QUALITY MGMT: (Nurses received continued mentorship at the clinics post-training; Evaluation of clinical care via charts were reviewed monthly, feedback was provided in the event of poor site performance; and an exchange clinic system was initiated, whereby good performing clinic staff would assist poor performing clinics) 3. RESTRUCTURE: (Task-shifting was used to train CO in traditional physician skills, nurses in CO skills, and peer educators in nursing skills)	None	1. PENETRATION: 174 Clinical officers and 333 nurses were trained in HIV care and treatment; 131 CO and 120 nurses were trained in pediatric HIV care and treatment; 91 nurses were trained in triage 2. FEASIBILITY: Task-shifting was successfully implemented at healthcare facility
54	Perez et al. [97]	PMTCT (HIV therapy)	1. RESTRUCTURE: (PMTCT services were initiated at a rural healthcare facility) 2. EDUCATE: (Nurses and midwives were trained to provide NVP to pregnant mothers, provide HIV counseling, and HIV testing) 3. QUALITY MGMT: (Audits and regular monitoring were performed during intervention)	1. PENETRATION: 220 women received NVP tablet to take home; 2298 (93%) women benefitted from pre-test counseling; 93% of counseled women accepted HIV testing; Of the 159 deliveries at the facility, 111 reported taking NVP during labor and 114 reported their children receiving NVP; Only 16 HIV+ women refused follow-up at discharge 2. FEASIBILITY: Reasons for refusing testing were desire to consult partner first)	1. FEASIBILTY: PMTCT program was implemented in rural health facility 2. PENETRATION: 20 nurses and mid- wives were trained in Nevarapine delivery and HIV counseling; More specifically, 2 midwives and three PMTCT staff were trained in HIV testing)
55	Sanne et al. [98]	ART (HIV therapy)	1. EDUCATE: (HCWs received training on ART) 2. RESTRUCTURE: (Task-shifting ART therapy from physicians to nurses)	1. PENETRATION: Primary study end- point was reached by 371 (45.7%) of patients; 192 (48%) were in the nurse arm, 179 (44%) were in the physician-arm; CD4 counts increased in both nurses and physician arms, but was slightly higher in nurse-arm at end of 2-year study; Baseline CD4 was 155 cells and 158 cells for nurses and physicians respectively, whereas, 239 cells and 220 cells for nurse and physicians was assessed at the study’s end 2. FEASIBILTY: Non-inferiority of nurse-initiated ART was assessed	1. PENETRATION: 812 patients enrolled in trail: 408 in nurses arm; 404 in physician arm; 2 nurses trained in ART
56	Ssekabira et al. [99]	Malaria screening, diagnosis, treatment	1. PLAN: (Ugandan Malaria Surveillance Project (UMSP) and the Malaria Control Program (MCP) established a surveillance system in 2006 to capture patient data; Malaria training materials were developed via a joint partnership between Joint Uganda Malaria Training Program (JUMP), UMSP, and Infectious Disease Institute (IDI) of Mekerer University) 2. EDUCATE: (HCWs were trained in malaria screening, diagnostics, and treatment; Training materials were developed via joint partnerships) 3. RESTRUCUTRE: (Malaria surveillance system was established)	1. PENETRATION: Post-training, the proportion of patients suspected of having malaria referred for microscopy increased by 50%; Proportions of patients with a negative blood smear prescribed antimalarials decreased by 60%; The proportion of decrease in inadequate antimalarial prescribing resulted in 8151 fewer prescriptions among the 67,705 patients over the 120 days after training	1. PENETRATION: 170 HCWs were trained in malaria screening, diagnostics, and treatment
57	Sserwanga et al. [100]	Malaria screening/surveillance/ intensive case finding	1. PLAN: (Partnership established among UMSP, MOH, and the Ungandan National Malaria Control Program (NMCP) to initiate the implementation of a malaria surveillance system) 2. EDUCATE: (HCWs were trained in malaria screening/surveillance; Training materials were developed via joint partnerships) 3. RESTRUCUTRE: (Malaria surveillance system was established and implemented at 6 sites; A UMSP team would visit sites every 1–2 months to ensure adequate supply of resources for testing) 4. QUALITY MGMT: (UMSP team would also provide feedback to HCWs at sites during monthly visits)	1. PENETRATION: 166,278 patients underwent diagnostic testing for malaria during the study period; Proportion of suspected patients who underwent diagnostic testing increased from 39% during the first three months of surveillance to 97% in the last three months of surveillance	1. PENETRATION: 84 nurses trained in malaria screening/surveillance/intensive case finding 2. FEASIBILITY: Malaria surveillance system was successfully implemented
58	Stringer et al. [101]	ART (HIV therapy)	1. PLAN: (A partnership among the Lusaka Urban Health District, the University Teaching Hospital, the Zambian National AIDS Council, and the Centre for Infectious Disease Research, and the University of Alabama was established) 2. RESTRUCTURE: (During 18-month study period, ART program was scale-up to 14 additional sites in Lusaka; Renovation of healthcare facilities to better be equipped to provide ART was undertaken; Form drive protocols were to be used during patient visits; EMR system was designed and implemented) 3. EDUCATE: (Training of non-physician clinicians occurred in ART; Clinical care oversight was provided to HCWs; Form-driven protocols were developed) 4. COST: (Non-physician clinicians were provided overtime; PEPFAR provided generous support for this study)	1. PENETRATION: Among 21,755 HIV+ treatment-naïve patients who were eligible for ART, 16,198 (74%) were started on ART; As of Nov. 2005, 11,591 (72%) of patients who had started ART remained alive and were continuing to take ART	1. PENETRATION: 29,998 HIV+ patients were evaluated; Using nurses in task-shifting capacities allowed the study/ART program to overcome the challenges of physician shortages, in short, nurse s helped get patients on ART; Rapid scale-up of ART was accomplished at 14 additional sites in Lusaka 2. ACCEPTABILITY: Zambian government provided substantial support of the scale-up ART strategy 3. COST: Significant funds from PEPFAR made this intervention possible
59	Umulisa et al. [102]	Hand hygiene/washing	1. RESTRUCTURE: (In stalling locally made hand hygiene facilities at point of patient care; ensuring availability of water at hand hygiene facilities) 2. EDUCATE: (HCWs received a 2-h training on hand hygiene; Posters were placed on the wards to serve as HCW reminders to wash hands) 3. QUALITY MGMT: (pre- and post-training observations were conducted on hand hygiene etiquette among HCWs; Supportive supervision was provided throughout training to ensure hand hygiene compliance)	None	1. ADOPTION: Ensuring hand gel and water were available at point of patient care was implemented at facility
60	Driessche et al. [103]	HIV Counseling/Testing AND TB testing/therapy	1. PLAN: (Stakeholder collaboration/partnership established for training development) 2.. EDUCATE: (Training materials were developed collaboratively by the DRC National HIV and TB Control Program officers, an education specialist, international TB experts, and HCWs; Trainings were conducted for HCWs on HIV/TB testing/counseling; Training was made dynamics via interactive Q&A sessions, case studies, group sessions, PP presentations, small breakout sessions) 3. QUALITY MGMT: (On-site supervisory visits and monthly follow-up meetings were used as a part of the training process; Feedback was provided during these supervisory sessions to HCWs; Revisions were made to the training manuals and materials post-intervention)	1. FEASIBILITY: Before training, the link between HIV and TB was unclear to some HCWs, such that only 67% of HCWs stated that there was a connection between the two diseases	1. ADOPTION: High rates of training participation were achieved (91% to 100%) for all training sessions 2. ACCEPTABILITY: Training received positive feedback from HCWs
61	Workneh et al. [104]	ART (HIV therapy)	1. PLAN: (A Center of Excellence was established between Baylor University and Botswana; COE developed mentorship program for HCWs) 2. EDUCATE: (On-site mentorship was provided to HCWs; Didactic sessions that were focuses on pediatric HIV care and treatment was also conducted)	1. FEASIBILITY: 6 out of the 14 clinical HIV indicators (i.e., ART dosing) had significant documentation to report on	1. ADOPTION: High rates of training participation were achieved (91% to 100%) for all training sessions 2. ACCEPTABILITY: Training received positive feedback from HCWs 3. FEASIBILITY: Retrospective chart review was conducted on 374 charts at four of the mentored sites

*Represents strategies that targeted nurses and/or measured outcomes associated with nurses

**Table 5:** Study characteristics associated with implementation strategies and outcomes for nurses and non-nurses (*N* = 61)

	Number of studies (*n*)	Percentage of studies (%)	Number of studies with strategy/outcome focused on nurses (*n*)	Percentage of studies with strategy/outcome focused on nurses (%)	Number of studies with strategy/outcome focused on patients or overall health system (*n*)	Percentage of studies with strategy/outcome focused on patients or overall health system (%)
Implementation strategies						
Education	59	96.7	58	95.1	1	1.6
Quality management	39	63.9	21	34.4	18	29.5
Plan	33	54.1	12	19.7	21	34.4
Restructure	32	52.5	7	11.5	25	41.0
Finance	8	13.1	2	3.3	6	9.8
Policy	0	0.0	0	0.0	0	0.0
Implementation EBP outcomes						
Penetration	22	36.0	4	6.5	18	29.5
Feasibility	13	21.3	6	9.8	7	11.5
Adoption	5	8.2	1	1.6	4	6.6
Sustainability	5	8.2	2	3.3	3	4.9
Acceptability	4	6.6	3	4.9	1	1.6
Fidelity	3	4.9	2	3.3	1	1.6
Appropriateness	1	1.6	1	1.6	0	0.0
Cost	0	0.0	0	0.0	0	0.0
No EBP outcome	21	34.4	na	na	na	na
Implementation strategy outcomes						
Penetration	44	72.1	34	55.7	7	11.5
Feasibility	28	45.9	6	9.8	22	36.1
Acceptability	18	29.5	13	21.3	5	8.2
Adoption	5	8.2	2	3.3	3	4.9
Sustainability	3	4.9	3	4.9	0	0.0
Appropriateness	2	3.3	2	3.3	0	0.0
Cost	2	3.3	2	3.3	0	0.0
Fidelity	0	0.0	0	0.0	0	0.0
No strategy outcome	3	4.9	na	na	na	na

### Implementation outcomes

For this review, the outcomes of the studies have been analyzed into two parts: outcomes associated with the EBPs and outcomes related to the implementation strategies used to promote an IPC EBP.

#### Implementation outcomes for EBPs

All implementation outcomes, except cost, were measured in the studies of this review. Most studies reported penetration (*n* = 22, 37%) and feasibility (*n* = 13, 21%) as the most common outcomes produced (see Table 5). Four (7%) and 18 (30%) studies measured penetration for nurses and non-nurses respectively (see Table 5). For the non-nurse category, penetration was measured as patient agreement to initiate an EBP (i.e., screening for TB or antiretroviral therapy (ART) uptake). Feasibility was frequently measured as nurse report that EBPs could be implemented within their scope of work. Additionally, feasibility was used to report barriers to EBP implementation. For example, limited hospital infrastructure (i.e., no running water) was identified as a limitation to hand hygiene adherence among nurses.

Few studies measured adoption (*n* = 5, 8%) and sustainability (*n* = 5, 8%). Adoption was discussed as patient willingness to accept an EBP with nurse support, facility-level uptake of an EBP, or nurse report of healthcare improvements as a result of an implemented EBP. Sustainability was measured as nurses’ ability to maintain an EBP for a few weeks or longer. Four (7%) studies discussed acceptability. Fidelity was also discussed in three (5%) studies. Fidelity was reported as adherence to an EBP by nurses. One (2%) study addressed appropriateness, and zero (0%) studies reported cost. Lastly, 21 (35%) studies did not measure any EBP outcome. In these studies, the outcomes of interest were related to the implementation strategies. Table 4 provides a complete list of implementation outcomes associated with EBPs for each study. Nurse-specific outcomes associated with EBPs are bolded in Table 4.

#### Implementation outcomes for implementation strategies

The most common outcomes measured for implementation strategies used in this review were penetration and feasibility; a similar finding to the outcomes associated with EBPs. Forty-four (72%) studies reported penetration. Thirty-four (56%) and seven (12%) studies reported penetration outcomes for nurses and non-nurses respectively (see Table 5). For nurses, penetration was most frequently used to measure the number of nurses experiencing or undergoing a given implementation strategy (e.g., number of nurses trained in hand hygiene). For the non-nurse category, penetration was used to scale-up patient services or measure loss to follow-up (see Table 5). Feasibility was measured in 28 (46%) studies, with six (10%) and 22 (36%) studies measuring feasibility for nurses and non-nurses respectively. Feasibility measured if an IPC protocol could be successfully implemented for nurses. Four studies successfully implemented the WHO hand hygiene campaign to promote hand washing for healthcare workers [32, 58, 80, 81], demonstrating that the intervention could be implemented. Feasibility also described barriers to the successful implementation of an implementation strategy or to the procurement of resources for IPC protocol implementation. Soap, alcohol-based hand gels, and towels were procured for nurses to eliminate barriers related to hand disinfection, making it feasible for nurses to implement the EBP. The third most common outcome reported was acceptability (*n* = 18, 30%). Acceptability was measured in 13 (21%) and five (8%) studies for nurses and non-nurses respectively (see Table 5). For nurses, this outcome measured nurses’ opinions on the strategy and its utility. Five (8%), three (55%), and two (3%) studies addressed adoption, sustainability, and appropriateness respectively. Sustainability captured nurses’ ability to sustain knowledge acquired during a training for a defined period of time. Appropriateness measured nurses’ perceptions of the cultural relevance of educational materials produced for a training or the benefit of training to the nursing profession. Cost, measured in only two (3%) studies, captured the amount of funds provided from the development sector to ensure that implementation strategies were carried out. Zero (0%) studies measured fidelity to implementation strategies.

### Quality appraisal

All studies (*n* = 61, 100%) included within this review met the pre-determined or global quality criteria. Only one study, which was a multi-modal study, had a score under the cut-off criteria for its quantitative section. The qualitative score for this study met criteria, and thus was included in the review, which adhered to the pre-determined criteria for multi-model studies. Fifty-five studies (90%) were quantitative or multi-modal studies. The most common MAStARI score was 5 (*n* = 19, 31%). The structure of the MAStaRI scales favors experimental designs; five articles with MAStARI scores of 9 or higher were randomized controlled trials. Three (4.9%) studies were purely qualitative, both examining the feasibility of an EBP implementation strategy. Three (4.9%) studies were mixed methods, and both achieved MMAT rating of 50% or higher.

## Discussion

Infectious diseases contribute significantly to patient and healthcare worker morbidity and mortality in SSA. Nurses who engage in direct patient care are at increased risk of nosocomial and other infections [22, 105]. To address these HAI challenges, many IPC EBPs have been deployed at the healthcare facility level. Yet, obstacles within LMICs make it difficult to sustain these practices. Implementation science provides an opportunity to identify and execute strategies that may better sustain EBPs over time [106]. Based on the results of this review, it is clear that *implementation* of IPC EBPs in SSA requires additional research, practice, and funding. More research from an explicit implementation science-specific perspective is needed for the following reasons: (1) very few of the studies in this review were able to produce sustainable outcomes, a potential limitation of the strategies implemented. Yet, more research is needed to know if this is true or not. (2) All studies in this review, except for one, did not identify as an implementation study. Thus, the strategies were not measured using implementation science definitions for strategies. Therefore, increased implementation science research into which, what combination, and in what specific context implementation strategies should be used in SSA is important to advance the science and to fully reap the potential of IPC protocols to save the lives of patients and healthcare workers in vulnerable regions.

Despite increased risk of contracting infectious diseases, nurses were underrepresented in the studies in this review. The majority of studies did not exclusively focus on nurses; they focused on non-nurse participants, including physicians, patients, auxiliary hospital staff, or some other aspect of the healthcare system, and nurses were included in the samples as generic healthcare workers. In studies where nurses were the target population, the numbers of nurses included in the study was often not reported or too low to draw generalizable conclusions. Given this finding, a shift is needed in global health research to properly determine what IPC EBP practices are best suited for nurse implementation at health facilities in SSA**,** especially as the tasks of nurses are different from other healthcare workers. Whereas there can be some lessons learned from other healthcare cohorts, the scope of practice for nurses, as it relates to IPC, is vastly different from other healthcare workers. While many different types of health care workers (including laboratory technicians and water and sanitation staff) are at increased risk of acquiring HAIs, this study focused on nurses for the following reasons: (a) nurses have unique needs (they spend the most amount of time with patients than any other health worker and operate in highly unstandardized and variable circumstances); (b) nurses are by far the largest cadre of the workers in SSA (even though their needs often take second place or are lumped to those of physicians). As infectious diseases evolve and become more prevalent, EBPS for IPC will become an increasingly important part of nurses’ scope of practice. Thus, additional and better-quality research that targets the unique IPC EBP challenges of the different healthcare worker cohorts, especially nurses and their involvement in IPC efforts, are needed.

Standard and transmission-based precautions were underemphasized in this review; most studies focused on administrative precautions. Standard precautions represent standard care. Therefore, an IPC study without a standard precautions component overlooked a critical component of IPC. Transmission-based precautions are typically undertaken when a patient is already diseased and to prevent further disease. Very few studies focused on this specific set of IPC EBPs, despite the fact that so many diseases in SSA, like TB, require them. Based on the results of this review, transmission-based and standard precautions are not sufficiently studied within LMICs, leaving a significant gap in the IPC literature.

The implementation strategies identified in this review lacked diversity. Of the seven implementation strategies identified, the predominant method was nurse education, specifically training on an IPC protocol. Johnson et al. [107] found similar results in a systematic review assessing implementation strategies for cervical cancer in SSA. Rowe et al. [108] argue that training in conjunction with other strategies (i.e., group problem solving, management techniques, supervision, and sound infrastructures) could improve healthcare worker practices in LMICs. Implementation science, along with other disciplines, acknowledges that access to information alone is not sufficient to produce long-term change [109]. Education only strategies are not likely to produce sustainable IPC knowledge or practice change among nurses. In addition to a lack of diversity in implementation strategies, there were notable gaps: policy and finance strategies, which play an important role in overall implementation, were rarely utilized. Unfortunately, the degree to which these strategies influence each other and other strategies (i.e., quality management) is relatively unknown given gaps in implementation science literature.

Among the studies in this review, it was identified that implementation strategies for nurses compared to non-nurses are vastly different. Education and quality management strategies are predominately used for nurses, and planning and restructure strategies are most often used for non-nurses. Nurses are trained and undergo regular monitoring, whereas non-nurses, especially patients, benefit from restructured clinical services and planned healthcare activities. Additional scholarship is needed to affirm if these strategies are indeed the best for each population.

Studies in this review that utilized multi-faceted implementation strategies produced better outcomes (such as feasibility, acceptability, etc.) compared to their single-strategy counterparts; a finding consistent with the current implementation science literature [110]. A comprehensive implementation approach, addressing potential challenges at each level of a healthcare system, yields stronger and more sustainable outcomes. Training, an educational strategy, and real-time audit/feedback, a quality management strategy, produced better outcomes when used together than alone [54]. More specifically, hand hygiene rates increased (8.0% to 21%, *p* < .001) when multiple strategies (quality management, education, planning) were used to promote this EBP, whereas hand hygiene rates declined (68.9% to 36.8%, *p* < .001) when only one strategy was used (quality management) [32, 33]. Multi-faceted strategies also generally focus on more than one stakeholder. Many of the studies in this review that used multi-faceted strategies targeted patients and healthcare workers. Single strategies focused on patient outcomes, perpetuating the underrepresentation of nurses, and overlooking nurse outcomes. Restructure is an excellent example of single strategy utilization. Most restructure strategies involved task-shifting nurse duties; however, this strategy is geared toward patient care and the impact on patient care, not the impact on the nurse practice. To advance implementation research within the global health context, multi-faceted strategies need to be promoted and better metrics need to be developed to capture outcomes related to multi-faceted approaches [111].

Many studies only reported on implementation strategy outcomes, not EBP outcomes. Two possible explanations for this finding are as follows: (1) most EBPs are already accepted as effective practices that do not require additional validation, and (2) measuring EBPs is challenging in a LMIC. Collecting data on an implemented strategy is fairly easy. Collecting and verifying EBP data is a significant undertaking in resource-constrained settings. However, implementation science research conducted in SSA must focus on some of the lesser reported outcomes, like cost or sustainability. Although these outcomes are more challenging to measure and require more time and funding to capture, implementation science studies that do not report on these outcomes have missed an opportunity to inform healthcare practices and policies at a macro-level.

Implementation outcomes measured for nurses compared to non-nurses are distinct. More EBP outcomes were measured for non-nurses, and more strategy outcomes were measured for nurses. The results of this review suggest that the relationship between EBP outcomes and non-nurses, specifically patients, may be related to how an EBP is defined. EBPs are “the conscientious, explicit and judicious use of current best evidence in making decisions about the care of the individual patient.” [112]. Patients, not nurses, are at the center of EBPs, which may explain the observed relationship between EBP outcomes and non-nurses. Educating patients is at the heart of the nursing profession; however, nurses need access to specific IPC EBPs information that is unique to them. Within global health, a common reality is that there are too few nurses to provide patient care. Investing in nurses and their safety must start to be a priority of the global community. If sources of inertia around nurse investment are not addressed within LMICs, then entire global communities will continue to suffer poor health as a result.

Studies with similar implementation strategies often used similar outcomes to measure impact. For example, the implementation of education interventions was often measured using an indicator of penetration (e.g., a training is implemented and researchers measured number of nurses trained). Similarly, planning strategies measured stakeholder response to an intervention. These outcomes were often not informative. A potential reason for this limited outcome data is related to the funding mechanisms of global health research and the types of outcomes that donors prioritize. The development sector is primarily interested in penetration outcomes (i.e., counts or proportions of nurses trained). In LMICs, purchasing resources for a project or building a new health facility is relatively achievable. It is much harder to ensure that resources and expertise are being used appropriately, health facilities have an adequate number of staff, and that overall operations of these facilities are sustainable. The “collect and measure what is easiest” notion is not going to improve the promotion of IPC EPBs in LMICs. Different outcome-strategy combinations need to be researched, allowing for increased growth of the implementation science discipline and increased IPC innovation in LMICs.

This review is not without limitations. The overall quality of the studies in this review was highly variable. Many studies were inadequately designed to capture outcomes of interest, biased, or were neither generalizable nor reproducible. Many studies only reported descriptive statistics. Many studies poorly described their methodologies. Additionally, most of the studies in this review were only just above the cut-off criteria for inclusion.

## Conclusion

This review provides evidence that additional research from an implementation science-specific perspective is needed to promote IPC protocols for nurses in SSA. While many of the studies included in this review did not frame their methods or outcomes as implementation-focused, they indeed were. Implementation science should be fostered and promoted in global health. While many IPC EBPs are well-known, the best strategies to successfully implement them remains undetermined for LMICs. Implementation science has a unique opportunity to expand scholarship around best implementation practices in SSA. For example, a better understanding of which strategies are best suited for specific contexts or phenomena in SSA, like an infectious disease epidemic, to promote IPC protocols is needed. In short, additional implementation science research could greatly contribute to the limited knowledge base around which strategies should be utilized at the onset and during an infectious disease epidemic to promote IPC protocols and keep nurses from becoming patients during these emergency situations. Furthermore, implementation science-specific research could also alter the way scholarship in SSA is prioritized and funded, especially as it pertains to development mechanisms. Given the region’s reliance on foreign assistance to cover healthcare expenditures [113], implementation science, with an emphasis on cost and policy, could identify investments for SSA and its healthcare workers.

Prevention is a critical component of infection control. The cost of IPC inaction is large. For the countries most affected by Ebola in 2014 (Sierra Leone, Liberia, and Guinea), economic modeling projections for post-epidemic healthcare strengthening initiatives estimate the cost of reconstruction to be US$877 million in 2018 [114]. These funds are necessary for infrastructure and service delivery changes, including IPC measures. With a stronger health system able to withstand a future epidemic, the loss of life, particularly of nurses, during the 2014 outbreak may have been avoided. With emerging infections currently challenging the global community, like the EVD epidemic in the DRC and Marburg virus identified in Sierra Leone for the first time [115], neglecting IPC prevention strategies, especially for nurses, is unaffordable.

## Supplementary information

**Additional file 1.** Key Search Terms and Search Results by Search Engine. (DOCX 20 kb)

## Data Availability

All data generated, produced, or analyzed for this study are included in this published article (including within supplementary files).

## Abbreviations

ART: Antiretroviral therapy
DALY: Disability-adjusted life year
EBP: Evidence-based practice
EVD: Ebola viral disease
HCAI: Healthcare-associated infections
IPC: Infection Prevention and Control
JBI: Joanna Briggs Institute
LMIC: Low-middle-income country
MAStARI: Meta-analysis for Statistics Assessment and Review Instrument
MMAT: Mixed methods appraisal tool
PRISMA: Preferred Reporting Items for Systematic Review and Meta-Analysis
SSA: Sub-Saharan Africa
TB: Tuberculosis
WHO: World Health Organization
