# Supplementary material for: Implementation strategies for infection prevention and control promotion for nurses in Sub-Saharan Africa: a systematic review
Source: Implement Sci. 2019 Dec 30;14:111. doi: 10.1186/s13012-019-0958-3 (PMC6937686; doi:10.1186/s13012-019-0958-3)
Supplement: Supplementary file 1 — Additional file 1. Key Search Terms and Search Results by Search Engine. (DOCX 20 kb) [file 13012_2019_958_MOESM1_ESM.docx]

**Additional File 1: Key Search Terms and Search Results by Search Engine**

| **Key Search Terms** | **Search Engines** | | | | | **Total** |
| --- | --- | --- | --- | --- | --- | --- |
|  | **Ovid/ Medline** | **Pubmed** | **CINHAL** | **Embase** | **Cochrane** |  |
| "Nurses"[Mesh]) OR "Licensed Practical Nurses"[Mesh]) OR "Nursing"[Mesh]) OR "Nursing Care"[Mesh]) OR "nursing" [Subheading]) OR "Nursing Staff"[Mesh]) OR "Students, Nursing"[Mesh]) OR "Midwifery"[Mesh]) OR "Nurse Midwives"[Mesh]) OR "Nurse Clinicians"[Mesh] OR "Nurse Specialists "[Mesh] OR "nurses" OR "licensed practical nurses" OR "nursing care" OR "nursing" OR "nursing staff" OR "nursing students" OR "midwifery" OR "midwives" OR "midwife" OR "nurse midwives" OR "midwifery students" "nurse specialists" OR "nurs*" OR "midwi*" | 657901 | 846,111 | 1292268 | 1024818 | 42686 | 3863784 |
| "Africa South of the Sahara"[Mesh] OR "sub-Sahara*" OR "Sub Sahara*" OR "Angola" OR "Benin" OR "Botswana" OR "Burkina Faso" OR "Burundi" OR "Cameroon" OR "Cape Verde" OR "Central African Republic" OR "Chad" OR "Comoros" OR "Republic of the Congo" OR "Democratic Republic of the Congo" OR "Cote d'Ivoire" OR "Djibouti" OR "Equatorial Guinea" OR "Eritrea" OR "Ethiopia" OR "Gabon" OR "The Gambia" OR "Ghana" OR "Guinea" OR "Guinea-Bissau" OR "Kenya" OR "Liberia" OR "Madagascar" OR "Malawi" OR "Mali" OR "Mauritania" OR "Mauritius" OR "Mozambique" OR "Namibia" OR "Niger" OR "Nigeria" OR "Rwanda" OR "Sao Tome and Principe" OR "Senegal" OR "Seychelles" OR "Sierra Leone" OR "Somalia" OR "South Africa" OR "South Sudan" OR "Sudan" OR "Swaziland" OR "Tanzania" OR "Togo" OR "Uganda" OR "Zambia" OR "Zimbabwe” | 375129 | 478755 | 47034 | 668,508 | 17931 | 1587357 |
| COMBINED terms (nurses AND Africa) | 9062 | 13058 | 15780 | 19700 | 1406 |  |
| "Infection Control"[Mesh]) OR "Infectious Disease Transmission, Patient-to-Professional"[Mesh]) OR "Infectious Disease Transmission, Professional-to-Patient"[Mesh]) OR "Primary Prevention"[Mesh]) OR "prevention and control" [Subheading]) OR "Universal Precautions"[Mesh]) OR "Hand Hygiene"[Mesh]) OR "Hand Disinfection"[Mesh]) OR "Needles"[Mesh]) OR "Needlestick Injuries"[Mesh]) OR "Medical Waste Disposal"[Mesh]) OR "Decontamination"[Mesh]) OR "Hand Sanitizers"[Mesh]) OR "Sterilization"[Mesh]) OR "Antisepsis"[Mesh]) OR "Personal Protective Equipment"[Mesh]) OR "Protective Devices"[Mesh]) OR "Respiratory Protective Devices"[Mesh]) OR "Cross Infection"[Mesh]) OR "Quarantine"[Mesh]) OR "Patient Isolation"[Mesh]) OR "Hospitals, Isolation"[Mesh]) OR "Triage"[Mesh]) OR "Contact Tracing"[Mesh] OR "infection control" OR "infection prevent*" OR "infection control and prevention" OR infectious disease transmission" OR "infectious disease control" OR "primary prevention" OR "prevention and control" OR "infection transmission" OR infection containment" OR "infection precautions" OR "pathogen prevent*" OR "pathogen control" OR "transmission precautions" OR "transmission-based precautions" OR "airborne precautions" OR "droplet precautions" OR "contact precautions" OR "universal precautions" OR "standard precautions" OR "hand hygiene" OR "hand disinfection" OR "hand decontamination" OR "handwashing" OR "hand washing" OR "gloves" OR "gown" OR "mask" OR "googles" OR "needles" OR "needlestick injury" OR "needlestick injury prevention" OR "needle stick injury" OR "medical waste disposal" OR "medical waste" OR "decontamination" OR "sterilization" OR "sanitation" OR "disinfection" OR "disinfectants" OR "antiseptics" OR "asepsis" OR "chlorine" OR "bleach" OR "hand sanitizer" OR "hand gel" OR "alcohol gel" OR "disinfect*" OR "sanitiz*" OR "steriliz*" OR "decontaminat*" OR "protective devices" OR "personal protective equipment" OR "protective equipment" OR "respiratory safety" OR "respiratory hygiene" OR "eye safety" OR "respirators" OR "N95" OR "cough hygiene" OR "cough etiquette" OR "cross infection" OR "nosocomial" OR "health-care associated infection" OR "health care associated infection" OR "health-care acquired infection" OR "health care acquired infection" OR "hospital acquired infection" OR "quarantine" OR "patient isolation" OR "isolation" OR "hospital isolation" OR "triage" OR "contact tracing" | 553502 | 1381819 | 437169 | 3335825 | 36749 | 5745064 |
| COMBINED terms (nurses/Africa/IPC) | 803 | 694 | 2683 | 3632 | 882 |  |
| ***COMBINED terms (nurses/Africa/IPC) (filter: English)*** | 755 | 664 | 2660 | 3529 | 792 | **8400** |
